# Mouse Transgenesis Identifies Conserved Functional Enhancers and *cis*-Regulatory Motif in the Vertebrate LIM Homeobox Gene *Lhx2* Locus

**DOI:** 10.1371/journal.pone.0020088

**Published:** 2011-05-23

**Authors:** Alison P. Lee, Sydney Brenner, Byrappa Venkatesh

**Affiliations:** Comparative Genomics Laboratory, Institute of Molecular and Cell Biology, A*STAR (Agency for Science, Technology and Research), Singapore, Singapore; Instituto de Medicina Molecular, Portugal

## Abstract

The vertebrate *Lhx2* is a member of the LIM homeobox family of transcription factors. It is essential for the normal development of the forebrain, eye, olfactory system and liver as well for the differentiation of lymphoid cells. However, despite the highly restricted spatio-temporal expression pattern of *Lhx2*, nothing is known about its transcriptional regulation. In mammals and chicken, *Crb2*, *Dennd1a* and *Lhx2* constitute a conserved linkage block, while the intervening *Dennd1a* is lost in the fugu *Lhx2* locus. To identify functional enhancers of *Lhx2*, we predicted conserved noncoding elements (CNEs) in the human, mouse and fugu *Crb2*-*Lhx2* loci and assayed their function in transgenic mouse at E11.5. Four of the eight CNE constructs tested functioned as tissue-specific enhancers in specific regions of the central nervous system and the dorsal root ganglia (DRG), recapitulating partial and overlapping expression patterns of *Lhx2* and *Crb2* genes. There was considerable overlap in the expression domains of the CNEs, which suggests that the CNEs are either redundant enhancers or regulating different genes in the locus. Using a large set of CNEs (810 CNEs) associated with transcription factor-encoding genes that express predominantly in the central nervous system, we predicted four over-represented 8-mer motifs that are likely to be associated with expression in the central nervous system. Mutation of one of them in a CNE that drove reporter expression in the neural tube and DRG abolished expression in both domains indicating that this motif is essential for expression in these domains. The failure of the four functional enhancers to recapitulate the complete expression pattern of *Lhx2* at E11.5 indicates that there must be other *Lhx2* enhancers that are either located outside the region investigated or divergent in mammals and fishes. Other approaches such as sequence comparison between multiple mammals are required to identify and characterize such enhancers.

## Introduction

*LIM homeobox gene Lhx2* is a member of the LIM homeobox family of transcription factors that are characterized by a LIM-type tandem zinc finger known as the LIM domain and a DNA-binding homeodomain. The *Lhx2* and its family member *Lhx9* are the vertebrate homologs of the fruit fly (*Drosophila*) *apterous* gene. *Apterous* is required for wing development, dorsoventral compartmentalization [1], [2] and neuronal pathway selection [3] in *Drosophila*. The murine *Lhx2* was identified through a screen for early markers of B-lymphocyte differentiation and determined to be involved in the differentiation of lymphoid and neural cell types [4]. *Lhx2*-null mice exhibit dorsal telencephalic patterning defects that involve an expansion of the choroid plexus and cortical hem at the expense of the hippocampus and neocortex [5], [6], ventral diencephalic defects that involve the infundibulum and pituitary gland [7], an absence of eyes [8], incomplete development of olfactory sensory neurons [9], liver fibrosis [10] and defective erythropoiesis resulting in death at E15.5 – E16.5 due to severe anemia [8]. Hence, *Lhx2* is essential for the normal development of the forebrain, eyes, olfactory system and liver. Recent studies have suggested that *Lhx2* acts as a classic “selector” gene that induces cortical stem cells to adopt hippocampal or neocortex identities [11]. *Lhx2* also plays an important role in maintaining hair follicle stem cells in an undifferentiated state [12], and the progression of anagen (growth phase) and morphogenesis of hair follicles [13].

In mouse, the expression of *Lhx2* begins at E8.5 in the optic vesicle [8], extending to a wide range of tissues by E10.5 including the telencephalon, diencephalon, optic cup, midbrain, hindbrain, future spinal cord [14] and liver [15]. At E11.5, *Lhx2* is localized in the walls of the lateral ventricles and third ventricle of the brain, the neural retina and optic stalk, the dorsal commissural interneurons of the neural tube and additionally expresses in limb bud mesenchyme [16]. By E15.5, *Lhx2* expression in the cerebral cortex becomes restricted to the ventricular layer and intermediate zone [5] and extends to the olfactory epithelium of the nasal cavity [17]. By E17.5, *Lhx2* expression in the cerebral cortex becomes restricted to the superficial layers of the entire cerebral cortex and the hippocampus (except for the subiculum) [5]. The restricted embryonic expression pattern of *Lhx2* is closely related to its function during development. For example, *Lhx2* has a graded expression pattern in the cortical ventricular zone (highest expression in the medial regions and lowest in the lateral regions) and is normally absent in the dorsal midline region [6]. This expression gradient is crucial for the role of *Lhx2* in specifying cortical cell fate, in particular by determining the regional fate (to either neocortex or olfactory cortex) in dorsal telencephalic progenitors [18]. In zebrafish, Six3, which is required for the formation of the entire rostral prosencephalon, acts upstream of Lhx2 suggesting that Six3 establishes the rostral forebrain field within which Lhx2 specifies cortical cell fate [11]. In *Xenopus*, transcription factors such as Pax6 and Six3 regulate the restricted expression of *Lhx2* in the developing eye. Together, these transcription factors form a gene regulatory network that helps specify the vertebrate eye field [19]. It has been proposed that *Lhx2* plays a central role in coordinating the various pathways that lead to optic cup formation [20]. Although the spatially and temporally restricted expression of *Lhx2* is crucial for proper development of the cerebral cortex and the eye, and that previous studies have indicated several potential upstream regulators of *Lhx2*, no attempt has been made to identify and characterize *cis*-regulatory elements in the *Lhx2* locus.

Human *LHX2* has been reported to be overexpressed in chronic myelogenous leukemia (CML) [21], but downregulated in small B-cell lymphoma [22] and lung cancer [23]. Little is known about the mechanisms by which changes in *LHX2* expression occur. The overexpression of *LHX2* in CML cells is postulated to be caused by decreased DNA methylation that results from a *BCR-ABL* gene fusion event [24] brought about by a translocation between chromosomal regions 22q11 and 9q34 [25]. However, *LHX2* (9q33.3) and *ABL* (9q34.12) are separated by a chromosomal distance as large as 7 Mb (human NCBI36 assembly). It remains to be seen if there exist *cis-*regulatory elements in the vicinity of *LHX2*, whose disruption could account for changes in *LHX2* expression.

In this study we have used evolutionary constraint as an indicator of putative enhancers in the vertebrate *Lhx2* locus. Due to selective pressure, noncoding functional elements such as enhancers tend to evolve slowly compared to their neighboring sequences and hence can be identified as conserved noncoding elements in comparisons of related genomes. This strategy has been effectively used to identify a large number of putative enhancers conserved in distantly related vertebrates such as mammals and teleost fishes [26], [27], [28], [29]. Functional assay of such elements in transgenic mouse and zebrafish have indeed indicated that a large number of them function as transcriptional enhancers directing tissue-specific expression of reporter genes during embryonic development [26], [28], [29]. We have aligned sequences of the *Lhx2* locus from human, mouse and pufferfish (fugu) genomes and predicted conserved noncoding elements (CNEs) in the locus. Functional assay of these CNEs in a transgenic mouse assay system showed that half of them function as tissue-specific enhancers at embryonic day 11.5.

## Methods

### Ethics statement

All animals were cared for in strict accordance with National Institutes of Health (USA) guidelines. The protocol was approved by the BRC Institutional Animal Care and Use Committee, Singapore (permit number 080338).

### Riboprobes for *in situ* hybridization

Primers that would produce 300-500 bp riboprobes were designed for murine genes *Lhx2*, *Crb2* and *Dennd1a*. PCR products were cloned into pBluescript II KS vector and sequenced to verify sequence identity and transcription orientation. The synthesis of digoxygenin (DIG)-labeled riboprobe was carried out based on published protocols [30] with the following modification: transcription was stopped by adding 1 µl of RNase-free DNase I (10 units/µl; Roche, Germany) and incubating at 37°C for 15 min. RNA concentration was in the range of 300–400 ng/µl. The riboprobes were designed to target the 5th (last), 7th and 22nd (last) coding exons of the mouse *Lhx2*, *Crb2* and *Dennd1a* genes respectively, and spanned 490 bp, 319 bp and 488 bp respectively.

### Whole-mount *in situ* hybridization

Whole-mount in situ hybridization of riboprobes was carried out as per published protocols [30], with the following modifications. For the Proteinase K step, concentration used was 10 µg/ml for 15 min; for hybridization, concentration of riboprobe used was 1 µg/ml; for post-hybridization washes, TBST instead of MABT was used; and for antibody incubation, embryos were incubated overnight in 10% heat-inactivated horse serum/1% blocking reagent (Roche, Germany)/TBST with 1∶2000 anti-DIG alkaline phosphatase-conjugated antibody (Roche, Germany). Before photo-taking, embryos were cleared in 50% glycerol/PBT and 80% glycerol/PBT (both times till embryos sunk). Pictures of the embryos were taken under 16× magnification using an Olympus SZX16 stereomicroscope (fitted with Olympus DP20 camera) and dark-field illumination.

### *Lhx2* locus sequence and alignment

Repeat-masked sequences for *Lhx2* locus from human, mouse and fugu genomes were downloaded from Ensembl release 48 (http://www.ensembl.org/) (human NCBI36 assembly, mouse NCBIM37 assembly and fugu v4.0 assembly). The sequences were aligned using MLAGAN [31]. CNEs were predicted using VISTA [32] based on the conservation criteria of ≥65% identity over 50 bp. Protein-coding and ncRNA sequences were identified and excluded based on searches against NCBI NR protein database, human and mouse EST databases (ftp://ftp.ncbi.nih.gov/blast/db/), Rfam (http://rfam.sanger.ac.uk/) and mirBase (http://www.mirbase.org/).

### Transgene constructs

Transgene constructs were prepared by linking CNEs upstream of a mouse *hsp68* minimal promoter (denoted as “*pHsp68*” in this report) and bacterial β-galactosidase reporter gene (*lacZ*). The *pHsp68-lacZ-pBluescript* vector was originally designed by Kothary et al. [33] and later modified by Nadav Ahituv (Lawrence Berkeley National Laboratory, USA) to incorporate a Gateway® cassette (attR1-*ccd*B-attR2) upstream of *pHsp68* for efficient cloning by Gateway® Technology (Invitrogen, USA). The *hsp68* minimal promoter alone has been shown to drive no *lacZ* expression in transgenic mice from E6.5 to E15.5 [33]. For PCR amplification of CNEs, primers were designed with 100–200 bp of additional flanking sequence on each side of the CNE with leading attB1/attB2 sequences for Gateway® DNA recombination. PCR was carried out using mouse genomic DNA as a template. ‘BP’ and ‘LR’ recombination reactions were carried out to clone the PCR product upstream of *pHsp68*. For both reactions, vector PCR was conducted to confirm that recombination was successful and the clones were sequenced to verify sequence identity. To generate constructs with base substitutions, QuikChange® Site-Directed Mutagenesis Kit (Stratagene, USA) was used.

### Transgenic mice

To prepare the transgene DNA for pronuclear microinjection, a restriction digest using *Sal*I (or double digest using *Hin*dIII and *Not*I) was performed to remove the *pBluescript* backbone. The transgene DNA was gel-purified (∼5 kb fragment) from vector backbone DNA (∼2.9 kb) using Geneclean® II Kit (Qbiogene, USA) and its concentration was estimated by running against known amounts of 1 Kb Plus DNA ladder (Invitrogen, USA). The DNA was diluted with filtered TE buffer (10 mM Tris pH 7.0, 0.1 mM EDTA pH 8.0) to a final concentration of 4 ng/µl and centrifuged twice at full speed for 15 min each to remove impurities, each time transferring 80% of the supernatant to a new tube. Transgenic mice were generated as per the standard protocol [34] using FVB/N mice as the host strain. DNA was extracted from the visceral yolk sac of embryos and used for genotyping by PCR using primers that amplify a 416-bp region which spans nucleotides 568 to 983 of the *lacZ* gene (GenBank accession number V00296) - *laczF*: 5′-CGT TGG AGT GAC GGC AGT TAT CTG-3′, *laczR*: 5′-CAG GCT TCT GCT TCA ATC AGC GTG-3′.

### Whole-mount *lacZ* staining

The expression of transgene was analyzed in E11.5 embryos by whole-mount *lacZ* staining as per published methods [35]. Before photo-taking, embryos were cleared in 50% glycerol/PBT and 80% glycerol/PBT (both times till embryos sunk).

### Expression analysis of human TF-encoding genes

GNF Human GeneAtlas v2 data (MAS5-condensed) were downloaded from GNF SymAtlas [36]. Expression values derived from cell lines and pathogenic tissues were removed, leaving behind expression values for 62 normal human tissues. To eliminate probes that showed very low expression and insufficient variance in expression levels across all tissues, probes with mean expression values less than 100 and standard deviation less than 50 were excluded [37]. Probe identifiers were mapped to human Ensembl gene identifiers using Ensembl BioMart (for HG-U133A probes) (http://www.ensembl.org/biomart/martview) and the annotation table from GNF SymAtlas (for GNF1H probes). Each gene's expression value in each tissue was computed by averaging expression values of all probes corresponding to that gene and normalizing the resulting value across 62 normal tissues such that mean expression is 0 and standard deviation is 1. Following this procedure, expression data were available for 17,210 human genes. *K*-means clustering was then performed on the normalized gene expression values with Cluster [38] and visualized with TreeView (http://rana.lbl.gov/EisenSoftware.htm). To test the alternative hypothesis that the average number (and length) of human-fugu CNEs associated with genes grouped into a cluster varies from that of genes grouped into other clusters, a Wilcoxon rank-sum test was applied. Two-tailed *P*-values were calculated.

### Motif finding

Statistically over-represented motifs were searched in human-fugu CNEs of TF-encoding genes using Weeder Version 1.3.1 [39]. Using oligomer frequencies in human intergenic regions as background, we searched both sequence strands for over-represented 8-mers with at most 2 substitutions, which appeared in at least 50% of the sequences and could occur more than once per sequence. Our search for 10-mers and 12-mers yielded no significant motifs. Interesting motifs with many close variants among the best 100 reported motifs were found using the program “adviser.out” in the Weeder package [39]. A chi-squared test (2-by-2 contingency table with Yates' correction for continuity; one-tailed *P*-values adjusted with Bonferroni correction) was used to determine which of these interesting motifs were over-represented (*P*<0.01) with respect to the complementary set of CNEs (e.g., CNEs of cluster #3 genes versus CNEs of non-cluster #3 genes).

## Results

### Organization of the *Lhx2* locus in vertebrates

**Figure 1:**
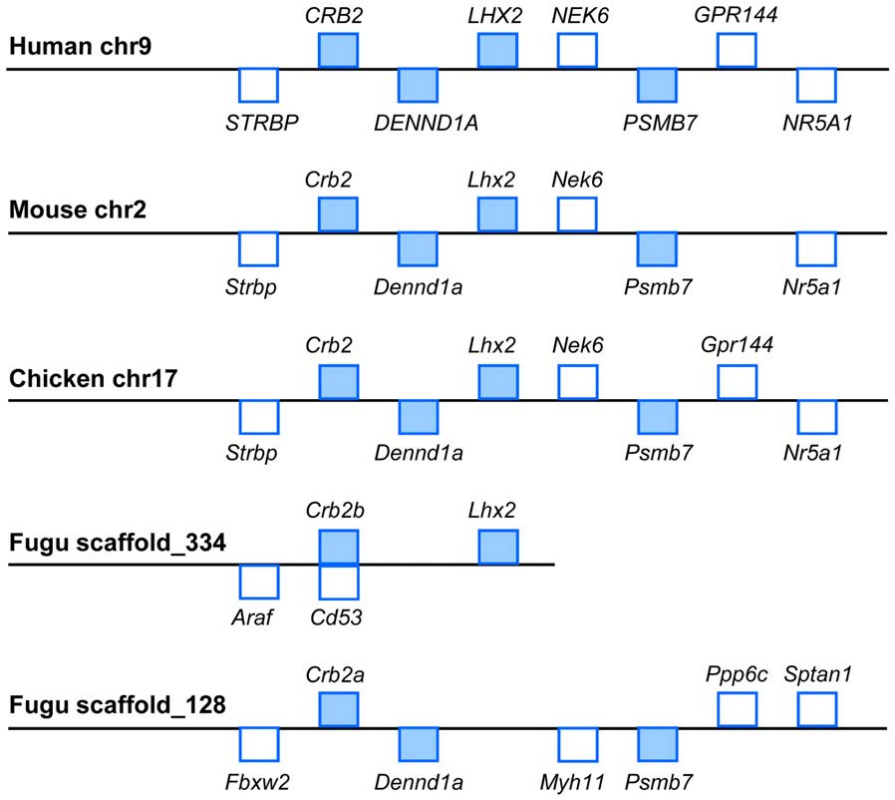
*Lhx2* gene loci in human, mouse, chicken and fugu. Rectangles above the line indicate genes on the forward strand, while rectangles below the line indicate genes on the reverse strand. Genes in blue are syntenic in the 4 species. Not drawn to scale.

We analyzed the *Lhx2* locus in the completely sequenced genomes of human, mouse, chicken and fugu. In human, mouse and chicken, the linkage of *Lhx2* and the two genes located upstream, *DENN/MADD-domain containing protein 1A* (*Dennd1a*) and *Crumbs homolog 2* (*Crb2*), is conserved (Figure 1). In fugu, *Lhx2* and *Crb2b* are syntenic on *scaffold_334* but *Dennd1a* is missing. Incidentally, the single remaining copy of *Dennd1a* in fugu is located on *scaffold_128* and is linked to *Crb2a*. In addition, the genomic sequence on fugu *scaffold_334* is incomplete ∼58 kb downstream of *Lhx2*.

The conserved synteny of the locus encompassing *Crb2* and *Lhx2* in vertebrates such as tetrapods and fish, led us to postulate that there may exist *cis-*regulatory elements in this syntenic block of *Crb2* and *Lhx2* that regulate the spatiotemporal expression of *Lhx2* and also *Crb2* and *Dennd1a* located upstream. As described by Kikuta et al. [40], genes that encode developmental TFs tend to be embedded in regions of extensive conserved synteny known as “genomic regulatory blocks”, and their *cis*-regulatory elements can be located far away, within or beyond neighbouring genes that are functionally unrelated. Based on this concept, it was necessary to examine the expression patterns of the adjacent genes *Crb2* and *Dennd1a* and investigate the possibility that *Crb2* and *Dennd1a* are regulated by CNEs within the *Crb2-Lhx2* conserved syntenic block. *Crb2* encodes two alternative isoforms, a transmembrane protein and a secreted protein, that are calcium-ion binding and are implicated in response to stimulus and visual perception. *CRB2* is expressed in adult human retina, brain, kidney, fetal eye and at lower levels in the lung, placenta and heart [41]. In zebrafish, there are two paralogs of the mammalian *Crb2*, *oko meduzy* (*ome*) and *crb2b*. The *ome* gene expresses in the developing brain and retina from 24–72 hpf and is essential for determining apico-basal polarity of neural tube epithelial cells [42]. The *crb2b* gene expresses in the immediate proximity of yolk sac and the pineal gland (epiphysis) from 24–48 hpf, and is highly enriched in the photoreceptor cell layer and pronephros at 72 hpf. It determines apical surface size in photoreceptors and is required for differentiation and motility of renal cilia [42]. Hence, because *Lhx2* and *Crb2* express similarly in the developing vertebrate brain and eye, there may also exist shared enhancers within the *Crb2-Lhx2* genomic region that direct expression of these two genes. On the other hand, *Dennd1a* encodes connecdenn, a component of the endocytic machinery with functions in synaptic vesicle endocytosis [43]. Connecdenn possesses an N-terminal DENN domain that is present in various signaling proteins involved in Rab-mediated processes or the regulation of MAP kinase signaling pathways [44], and other domains that enable it to bind clathrin adaptor protein 2 (AP-2) and synaptic Src homology 3 (SH3)-domain proteins. Recent work has shown that connecdenn's DENN domain acts as a guanine nucleotide exchange factor for Rab35 GTPase, enabling it to promote cargo-selective recycling from early endosomes [45]. The expression of connecdenn is preferentially high in the adult rat brain and testis, in particular the membranes of neuronal clathrin-coated vesicles [43], and relatively lower in liver, kidney, lung, heart and epididymis [46]. The expression domains shared with *Lhx2* (brain, liver) and *Crb2* (brain, kidney, lung, heart) might suggest that *Dennd1a* could be co-regulated with *Lhx2* and *Crb2*. However, *Dennd1a* has been lost from the *Crb2-Lhx2* conserved syntenic block in fugu, which implies that it does not fall under the control of *cis*-regulatory elements in the *Crb2-Lhx2* region.

### Expression patterns of *Lhx2*, *Crb2* and *Dennd1a* in E11.5 mouse embryos

**Figure 2:**
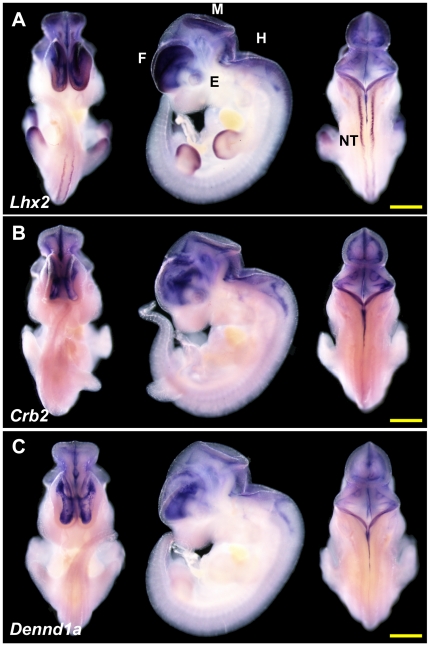
Expression of *Lhx2*, *Crb2*, *Dennd1a* in E11.5 mouse embryos. Expression patterns (ventral, lateral and dorsal views) as determined by whole-mount *in situ* hybridization at E11.5 for genes (A) *Lhx2*, (B) *Crb2*, and (C) *Dennd1a*. Three to four embryos were assayed for each gene and all embryos gave essentially the same results as these representative embryos. Scale bar at lower right corner denotes 1 mm in length. F: forebrain; M: midbrain; H: hindbrain; E: eye; NT: neural tube.

We determined the expression patterns of *Lhx2*, *Crb2* and *Dennd1a* genes in E11.5 mouse embryos by whole-mount *in situ* hybridization. While the expression pattern of mouse *Lhx2* at E11.5 has been previously been determined [16], [47], [48], to the best of our knowledge, there is no report of the expression patterns of *Crb2* and *Dennd1a* genes in E11.5 mouse embryos. Our *in situ* hybridization showed that at E11.5, *Lhx2* is expressed in the telencephalon, diencephalon, midbrain, hindbrain, eye, dorsal neural tube and distal regions of the limb buds (Figure 2A). This is in accordance with what has been previously reported about *Lhx2* expression [14], [16], [47]. *Crb2* and *Dennd1a* are expressed in the forebrain, midbrain, and hindbrain, which is strikingly similar to *Lhx2*. In addition, *Crb2* expresses in the eye. However, compared with *Lhx2*, both genes are expressed more highly in the medial regions of the telencephalon than in the lateral regions (Figure 2B and C) and do not express in the neural tube or the distal region of the limb buds.

### CNEs in the *Lhx2* gene locus

**Figure 3:**
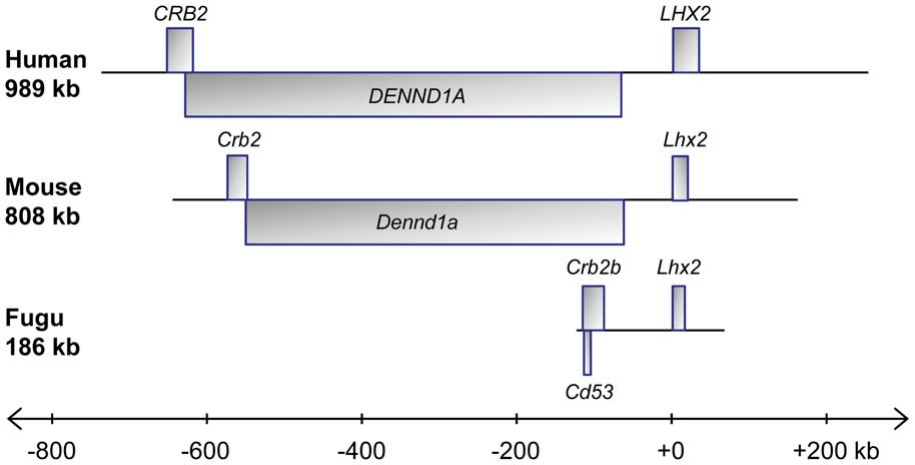
Region of conserved synteny in human, mouse and fugu used for sequence alignment. *Lhx2* locus in human, mouse and fugu used for sequence alignment. In human and mouse, *Crb2* and *Dennd1a* overlap by ∼100 bp at their 3′ ends.

**Figure 4:**
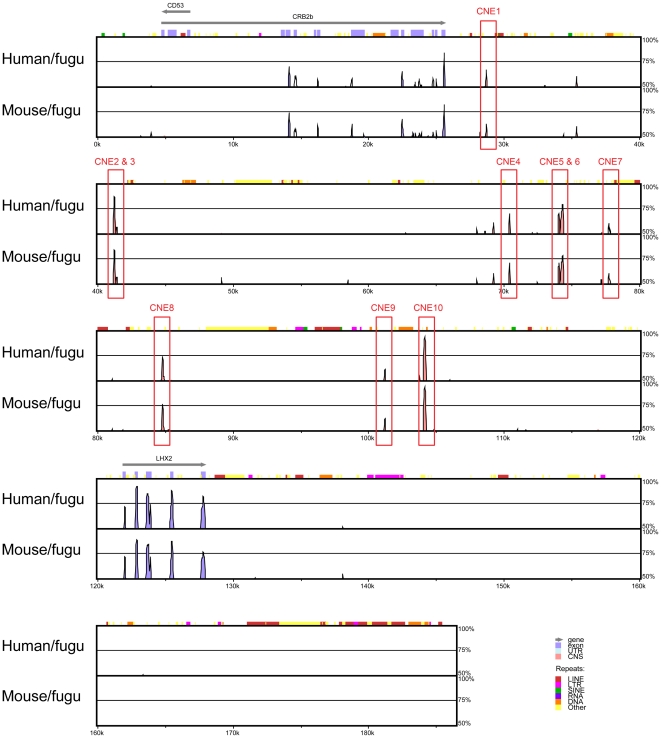
CNEs in the *Lhx2* locus. Human, mouse and fugu *Lhx2* loci were aligned using MLAGAN and CNEs (≥65% identity over 50 bp) were predicted with VISTA. Fugu served as the base sequence. Pink peaks denote CNEs while blue peaks denote conserved coding sequences. The arrows above the blue boxes that denote exons indicate the direction of transcription. *x*-axis represents distance along the fugu sequence while *y*-axis shows the percentage identity in each pairwise alignment. The pink peaks that are not annotated do not meet the thresholds of ≥65% identity over 50 bp.

**Figure 5:**
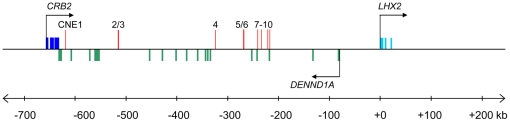
Location of CNEs in the human *LHX2* locus. The exons of protein-coding genes are shown in light/dark blue or green rectangles. The CNEs are located within the introns of the upstream gene *DENND1A* are indicated by red rectangles that extend above the exons.

To identify a comprehensive list of CNEs that may direct the expression of *Lhx2*, we aligned the entire region of conserved synteny beginning from the end of the gene upstream of *Crb2* to the start of the gene downstream of *Lhx2* in human and mouse and the end of *scaffold_334* in fugu (see Figure 1) using MLAGAN [31] and predicted CNEs using VISTA [32]. The length of this region is 989 kb in human, 808 kb in mouse and 186 kb in fugu (Figure 3). Ten human-fugu and mouse-fugu CNEs were identified using criteria ≥65% identity over 50 bp and were numbered *CNE1* to *CNE10* (Figure 4), beginning from the CNE that is located furthest from the transcription start site of *Lhx2*. The CNEs are 51 bp to 243 bp in length (average 115 bp), possess 65% to 90% sequence identity (average 72% identity) and reside 219 kb to 619 kb upstream of human *LHX2*, within the introns of the upstream gene *DENND1A* (Table 1; Figure 5).

**Table 1:** Details of CNEs tested in transgenic mice.

CNE construct	Length of CNE(s) (bp)	Percentage identity of CNE(s)	Distance from TSS of human *LHX2*	Location of CNE(s)
CNE1	76	69.1%	619 kb	*DENND1A* intron 20
CNE2/3	210	75.0%	516 kb	*DENND1A* intron 13
CNE4	119	71.4%	324 kb	*DENND1A* intron 5
CNE5/6	380	72.3%	269 kb	*DENND1A* intron 5
CNE7	51	64.7%	242 kb	*DENND1A* intron 3
CNE8	120	67.7%	234 kb	*DENND1A* intron 3
CNE9	53	69.8%	222 kb	*DENND1A* intron 3
CNE10	145	89.7%	219 kb	*DENND1A* intron 2

TSS: transcription start site.

### Functional assay of CNEs in E11.5 transgenic mouse embryos

The CNEs were amplified from mouse genomic DNA including 100–200 bp of flanking sequence on either end of the CNE, and cloned upstream of a *lacZ* reporter gene whose expression is driven by a mouse *hsp68* minimal promoter (“*pHsp68*”). Transgenic mouse embryos were generated and harvested at E11.5. The details of the CNE constructs tested in transgenic mice are given in Table 1 and Table S1. We studied the *lacZ* (β-galactosidase) expression patterns that were directed under the control of each CNE. A CNE was classified as a transcriptional enhancer if it directed reproducible reporter gene expression in the same anatomical structure in at least three independent transgenic mouse embryos.

### CNE1

*CNE1* is a 76-bp sequence located approximately 619 kb upstream of human *LHX2* within the 20^th^ intron of *DENND1A*. Altogether seven transgenic embryos were obtained for *CNE1* construct, of which four (57%) showed no *lacZ* expression while three (43%) showed varying patterns of *lacZ* expression (Figure S1). Although two independent embryos showed similar expression in the dorsal root ganglia (marked with red arrows in Figure S1), the number of transgenic embryos displaying this pattern of expression is not sufficient for this CNE to be classified as an enhancer. We therefore concluded that *CNE1* does not act as a transcriptional enhancer at stage E11.5.

### *CNE2/3*

**Figure 6:**
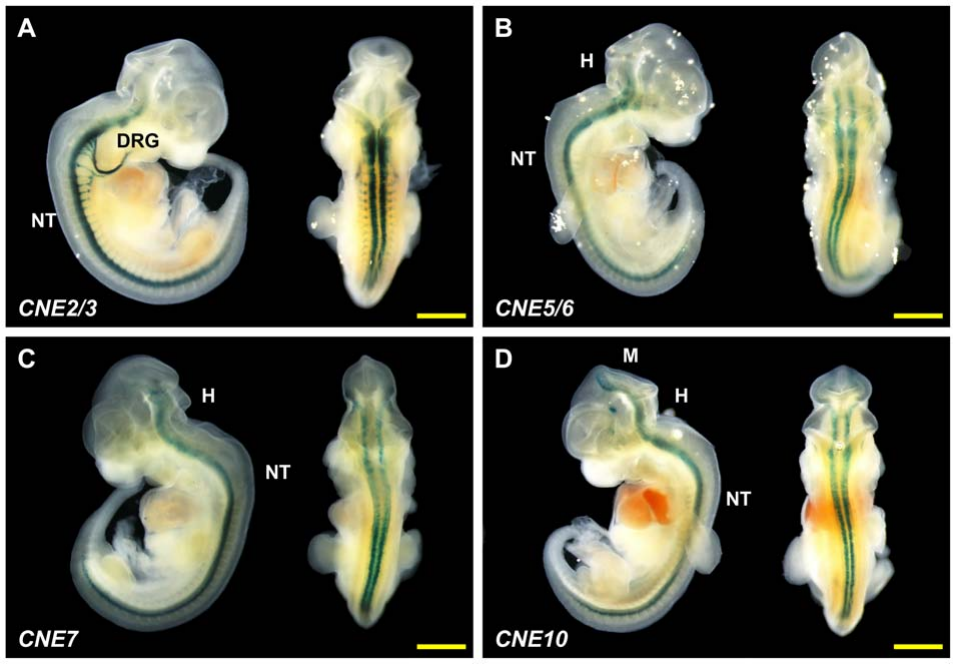
*Lhx2-*associated CNEs direct expression to various tissues in E11.5 mouse embryos. Lateral and dorsal views of a representative transgenic embryo for each construct. (A) CNE2/3; Strong *lacZ* expression was observed in the neural tube and dorsal root ganglia. (B) CNE5/6; (C) CNE7; *lacZ* expression was observed in the hindbrain and the neural tube for CNE5/6 and CNE7. (D) CNE10. *lacZ* expression extending from the most rostral region of the midbrain to the hindbrain and the entire length of the neural tube. Additional ectopic *lacZ* expression was detected in the diencephalon for this embryo. Scale bar denotes 1 mm in length. M: midbrain; H: hindbrain; NT: neural tube; DRG: dorsal root ganglion.

*CNE2/3* is a combination of two CNEs, *CNE2* and *CNE3*, which were tested together due to their close proximity to each other (41 bp apart). These CNEs have a combined length of 210 bp, and are situated approximately 516 kb upstream of human *LHX2* in the 13^th^ intron of *DENND1A*. A total of six E11.5 transgenic embryos were obtained for *CNE2/3* construct, four (67%) of which displayed reproducible expression in the neural tube and dorsal root ganglia (Figure 6A and Figure S2) while the remaining two embryos (33%) showed no expression. We therefore conclude that CNE2/3 directs expression to neural tube and dorsal root ganglia at E11.5. However, it should be noted that this expression pattern differs from *Lhx2* expression in the neural tube; while *lacZ* appears to express in the ventral region of the neural tube under the direction of *CNE2/3*, *Lhx2* expresses in the dorsal region at E11.5. Furthermore, the dorsal root ganglion is not a domain of *Lhx2* expression.

### *CNE4*

*CNE4* is a 119-bp element situated approximately 324 kb 5′ of *LHX2*, within the fifth intron of *DENND1A*. A total of five transgenic embryos were obtained for E11.5, but none of them showed *lacZ* expression (data not shown). Hence, *CNE4* is not an enhancer at E11.5.

### *CNE5/6*

*CNE5/6* is a combination of two CNEs, *CNE5* and *CNE6*, that are 38 bp apart and are located approximately 269 kb upstream of human *LHX2* within the fifth intron of *DENND1A*. A 719-bp genomic fragment was amplified and cloned for this construct, making it the longest element among all the constructs tested. A total of five E11.5 transgenic embryos were obtained. Four (80%) of these embryos had two *lacZ*-expressing anatomical features in common - the hindbrain and the neural tube (Figure 6B and Figure S3A – C) while the fifth embryo showed nearly ubiquitous expression throughout the entire head and the dorsal part of the embryo (Figure S3D). *CNE5/6* is classified as a transcriptional enfhancer at E11.5 because it directs reproducible reporter gene expression to similar embryonic regions in independent transgenic embryos. Although *CNE5/6* does not recapitulate the expression of *Lhx2* in the dorsal region of the neural tube, it does partially recapiftulate the expression of *Lhx2*, *Crb2* and *Dennd1a* in the hindbrain.

### *CNE7*

The 51-bp *CNE7* is the shortest among all the 10 CNEs tested. The orthologous sequences in human and fugu share 65% identity. *CNE7* is located in the third intron of *DENND1A*, approximately 242 kb upstream of *LHX2* in human. When assayed in transgenic mice, this construct directed reporter gene expression in the hindbrain and neural tube at E11.5. Among a total of four transgenic embryos that were obtained, three (75%) showed similar *lacZ* expression in the hindbrain and neural tube (Figure 6C and Figure S4) while the remaining embryo did not display any expression. Hence, *CNE7* acts as a transcriptional enhancer at stage E11.5. While *CNE7* does not recapitulate *Lhx2* expression in the dorsal region of the neural tube, it does partially recapitulate *Lhx2*, *Crb2* and *Dennd1a* expression in the hindbrain.

### *CNE8*

*CNE8*, like *CNE7*, resides in the third intron of *DENND1A*, approximately 234 kb upstream of human *LHX2* gene. This CNE does not act as an enhancer at E11.5, because no *lacZ* expression was detected in four out of the six (67%) transgenic embryos obtained for this developmental stage. The remainfing two embryos (33%) exhibited ectopic *lacZ* expression in various anatomical structures with no reproducible similarities (Figure S5).

### *CNE9*

*CNE9* is a 53-bp element, the second shortest CNE after *CNE7*, that is also located in the third intron of *DENND1A*, approximately 222 kb upstream of human *LHX2*. *CNE9* was amplified and cloned as part of a 261-bp insert fragment. When assayed in transgenic mice, a total of eight transgenic embryos were obtained, half of which showed no expression (data not shown) while three embryos showed ectopic expression in various tissues and the final embryo expressed *lacZ* ubiquitously (Figure S6). Hence, *CNE9* does not act as an enhancer at E11.5.

### *CNE10*

*CNE10* is a 145-bp element that has the highest percentage identity (∼90%) in human and fugu among all the CNEs assayed. *CNE10* is located approximately 219 kb upstream of human *LHX2*, immediately upstream of the third exon of *DENND1A*. Because *CNE10* encompasses a splice site of *DENND1A*, it may be involved in the regulation of *DENND1A* mRNA splicing. Nevertheless, we assayed this element for enhancer activity in transgenic mice because the ortholog of *DENND1A* is absent from the genomic region upstream of fugu *Lhx2* on *scaffold_334* suggesting that the CNE may have been retained during evolution due to selective pressure that arose from an underlying *cis*-regulatory function. Indeed, *CNE10* was found to act as a transcriptional enhancer. Of the total seven transgenic embryos obtained, three embryos (43%) showed reproducible *lacZ* expression in essentially the same anatomical features, the most rostral part of the midbrain, the hindbrain and the neural tube (Figure 6D and Figure S7) while two embryos showed ubiquitous *lacZ* expression throughout the whole embryo and the remaining two embryos did not display any *lacZ* expression. Although *CNE10* does not recapitulate *Lhx2* expression in the dorsal region of the neural tube at E11.5, it does partially recapitulate *Lhx2*, *Crb2* and *Dennd1a* expression in the midbrain and hindbrain.

### Summary of the expression patterns of CNEs

In summary, four out of the eight elements (50%) that we assayed for enhancer activity in transgenic mouse embryos, directed reproducible reporter gene expression in specific tissues at E11.5 (Table 2). Elements *CNE2/3*, *CNE5/6*, *CNE7* and *CNE10* directed *lacZ* expression to the neural tube with the latter three also directing *lacZ* expression to the hindbrain. Thus there was clear overlap in their domains of expression. In addition, *CNE2/3* induced *lacZ* expression in the dorsal root ganglia while *CNE10* extended *lacZ* expression from the hindbrain into the ventral and most rostral regions of the midbrain. Overall, these four elements partially recapitulate *Lhx2* expression in the midbrain and hindbrain, but do not recapitulate the expression of *Lhx2* in the forebrain, eye, limb buds and dorsal neural tube at E11.5.

**Table 2:** Summary of the expression patterns directed by CNE constructs.

	Number of mouse embryos					Reproducible expression pattern (if any)			
CNE construct	Transgenic	No *lacZ* expression	Ubiquitous expression	Ectopic expression	Reproducible expression	Midbrain	Hindbrain	Neural tube	Dorsal root ganglia
CNE1	7	4	-	3	-				
CNE2/3	6	2	-	-	4			•	•
CNE4	5	5	-	-	-				
CNE5/6	5	-	1	-	4		•	•	
CNE7	4	1	-	-	3		•	•	
CNE8	6	4	-	2	-				
CNE9	8	4	1	3	-				
CNE10	7	2	2	-	3	•	•	•	

### Prediction of motifs in CNEs associated with transcription factor-encoding genes that express in central nervous system

**Figure 7:**
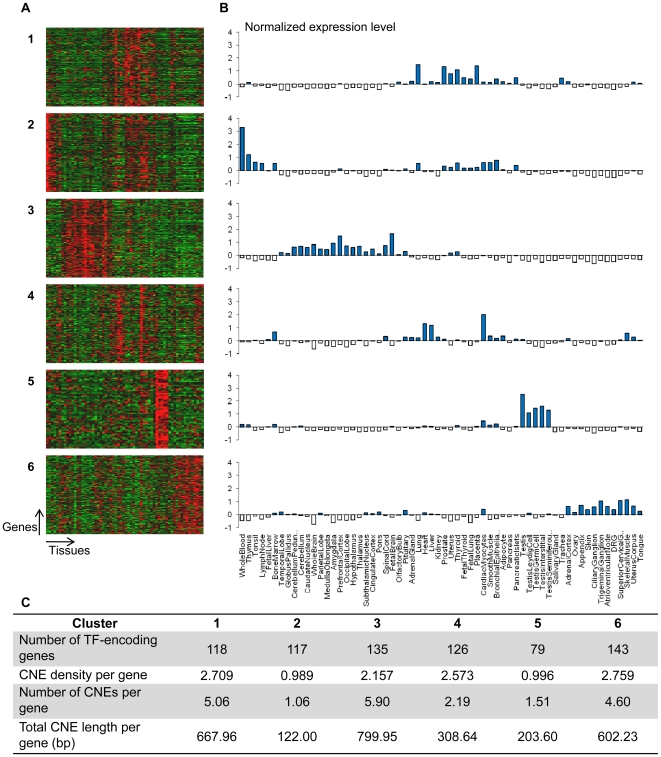
TF-encoding genes predominantly expressed in the central nervous system are enriched with CNEs. (A, B) Tissue expression patterns of 718 human TF-encoding genes. (A) Each of the six panels displays a heat map. The rows and columns of each heat map represent human TF-encoding genes and 62 human tissues respectively. (B) Each graph represents the average gene expression levels of a cluster of TF-encoding genes. Expression cluster #3 consists of genes that predominantly express in the central nervous system. (C) The table pertains to the CNE density (CNE bases per kb of noncoding sequence in a human gene locus), the number and total length of CNEs per TF-encoding gene in different expression clusters.

Transcriptional enhancers typically comprise clusters of binding sites for transcription factors (TFs). Since four of the *Lhx2*-associated CNEs (CNE2/3, CNE5/6, CNE7 and CNE10) function as enhancers directing expression to the central nervous system, we reasoned that they might be enriched for motifs (binding sites of TFs) that mediate expression in the central nervous system. Since four is a small number for predicting motifs, we decided to use the large set of human-fugu CNEs (∼3,000 elements that are at least 65% identical over 50 bp) that we have previously predicted in the TF-encoding gene loci in human and fugu [49]. This is a comprehensive set of CNEs associated with 718 human TF-encoding genes that have orthologs in fugu. We first extracted the expression data for 718 human-fugu TF orthologs across 62 human tissues from GNF Human GeneAtlas v2 [36]. The gene expression profiles were then clustered using the *k*-means clustering algorithm, Cluster [38]. We experimented with different values of *k* and selected the value *k* = 6 (resulting in six clusters) for subsequent analysis because it performed best qualitatively. Figure 7A shows the heat maps generated using TreeView (http://rana.lbl.gov/EisenSoftware.htm) and Figure 7B the corresponding average gene expression levels for each of the 62 human tissues across the six clusters. The CNE densities of genes in expression clusters #1, #3, #4 and #6 (Figure 7C) are higher than, but not significantly different (*P*>0.01; Wilcoxon rank-sum test with Bonferroni correction for multiple testing) from, those in other expression clusters. However, when the number of CNEs and total length of CNEs per gene are considered, genes in cluster #3 have a significantly higher number of CNEs (5.90 CNEs per gene, *P = *0.00983) than genes in other clusters, although their total length is not significantly high (total length 800 bp per gene, *P = *0.0124) (Figure 7C). This cluster consists mainly of genes that express highly in different regions of the brain (e.g., prefrontal cortex, amygdala, whole brain) and spinal cord, which make up the central nervous system. This suggests that TF-encoding genes that predominantly express in the central nervous system contain higher numbers of CNEs compared to TF-encoding genes that express in other tissues. This inference is in agreement with our previous findings that genes that are involved in development, in particular development of the central nervous system, are enriched with CNEs [49]. In addition, these results are consistent with the findings of Sironi et al. [50] that central nervous system-expressed genes tend to be rich in CNEs while Pennacchio et al. [29] who had verified ∼80 transcriptional enhancers in developing mouse embryos, found that the enhancers directed reporter expression most frequently in the brain and neural tube. More recently, a similar association between ultraconserved noncoding elements (noncoding sequences exceedingly conserved in human, chimp and mouse) and genes that preferentially express in the central nervous system has been described [51]. Using the GNF Human GeneAtlas, this study found that nine out of the ten tissues that express genes most significantly associated with ultraconserved noncoding elements constitute various members of the central nervous system.

The identification of a large number of CNEs (810 CNEs) associated with genes that express in the central nervous system (cluster #3) (Figure 7B) allowed us to search for over-represented motifs using the motif finding program, Weeder [39]. Comparison of CNEs associated with cluster #3 genes and non-cluster #3 genes (810 CNEs of 117 genes vs. 1,684 CNEs of 601 genes) resulted in the detection of four over-represented 8-mer motifs (*P*<0.01) (Table 3). These over-represented motifs in CNEs of cluster #3 genes presumably represent binding sites for TFs that are involved in directing expression of the target gene to the central nervous system. Interestingly, all the four motifs contain the “TAAT” motif characteristic of homeodomain TF binding sites (TFBS) [52]. Two of the motifs (motif #1, ‘ATTAACCG’ and motif #4, ‘TGATTACG’) coincide with two pentamer motifs (‘ATTAA’ and ‘GATTA’) previously reported to be enriched in four human-fugu CNEs that drove reporter gene expression in the mouse forebrain [29]. Motif #2 (‘CTAATTAG’) shares the ‘TAATT’ sequence of homeodomain TFBS, whose co-occurrence with SOX and POU TFBS in highly conserved mammal-fugu CNEs was proposed to be associated with gene expression in the central nervous system [53]. However, the motifs identified by Pennacchio et al. [29] and Bailey et al. [53] that coincide with our motifs, were not experimentally tested for functional activity in the central nervous system. Finally, a third study searched 13 forebrain enhancers conserved in human and zebrafish and identified five hexamer motifs that were enriched [54]. Mutation of the 5 motifs in some forebrain enhancers significantly reduced or altered enhancer activity and hence these motifs were deduced to be critical for forebrain enhancer activity. Our motif #3 (‘CATTAGCG’) partially corresponds to one of their motifs ‘AATGGA’.

**Table 3:** Over-represented 8-mer motifs in CNEs of cluster #3 genes.

				Known TFBS		
No.	Motif (and reverse-complement)	No. of instances	P-value	Sequence	TF	References
1	ATTAACCG (CGGTTAAT)	159	1.1×10^−12^	ATGYTAAT	Brn2	[64]
2	CTAATTAG (palindromic)	276	6.8×10^−8^	GCATAATTAAT	Brn3	[65]
				TAATTA	HoxB1, HoxB3	[66]
3	CATTAGCG (CGCTAATG)	98	5.2×10^−4^	CYYNATTAKY	HoxA5	[67]
				CATTAG	Isl-1	[68]
4	TGATTACG (CGTAATCA)	124	4.2×10^−3^	AATTAATCAA	Pit-1	[69]

Human-fugu CNEs of cluster #3 human transcription factor-encoding genes were compared against CNEs of non-cluster #3 transcription factor-encoding genes. Number of instances of each motif was determined by searching each motif in the human-fugu CNEs permitting at most 2 mismatches. *P*-values were calculated based on a chi-squared test followed by Bonferroni correction for multiple testing. Underlined portion of TFBS sequence indicates similarity with motif. TF, transcription factor; TFBS, TF binding sites.

We searched TRANSFAC for TFBS that matched instances of the four motifs in our 810 human-fugu CNEs associated with central nervous system-expressing genes. The identified sites included binding sites for homeodomain TFs such as HoxA5, Brain-2, Islet-1 and Pit-1 (Table 3) which are implicated in the development of the nervous system [55], [56], [57], [58]. Thus, the motifs we have found in the central nervous system CNEs are likely to be the binding sites for TFs that mediate expression of genes in the central nervous system. These motifs are putative targets for experimentally verifying TFBS in CNEs and for identifying upstream regulators of the associated TF-encoding gene.

Besides cluster #3, genes of clusters #1 and #6 also display relatively high numbers of CNEs although the association of these genes to presence of CNEs is not statistically significant. Cluster #1 genes are expressed highly in the human lung, placenta, prostate, thyroid and uterus (top five expression domains) (Figure 7B). To the best of our knowledge, these expression domains have not been previously associated with enrichment of CNEs. Cluster #6 genes are expressed highly in the human skeletal muscle, superior cervical ganglion, trigeminal ganglion, appendix and uterus corpus (top five expression domains) (Figure 7B). Among the top 10 expression domains, four tissues (superior cervical ganglion, trigeminal ganglion, adrenal cortex and ciliary ganglion) are derived from the neural crest. TF-encoding genes such as *Sox10* [59] and *Pax3* [60] are known to be associated with conserved enhancers that mediate gene expression in the neural crest or its derivatives. Despite having no significant enrichment of CNEs, the genes in the expression clusters other than cluster #3 may still be enriched for motifs that direct expression to the tissues characteristic of each cluster. To obtain a more complete list of motifs enriched in all TF-encoding genes, we searched the CNEs associated with the genes of the remaining expression clusters and identified two to twelve motifs enriched in CNEs of each cluster (Table S2).

### Site-directed mutagenesis of a predicted motif in *CNE2/3*

**Figure 8:**
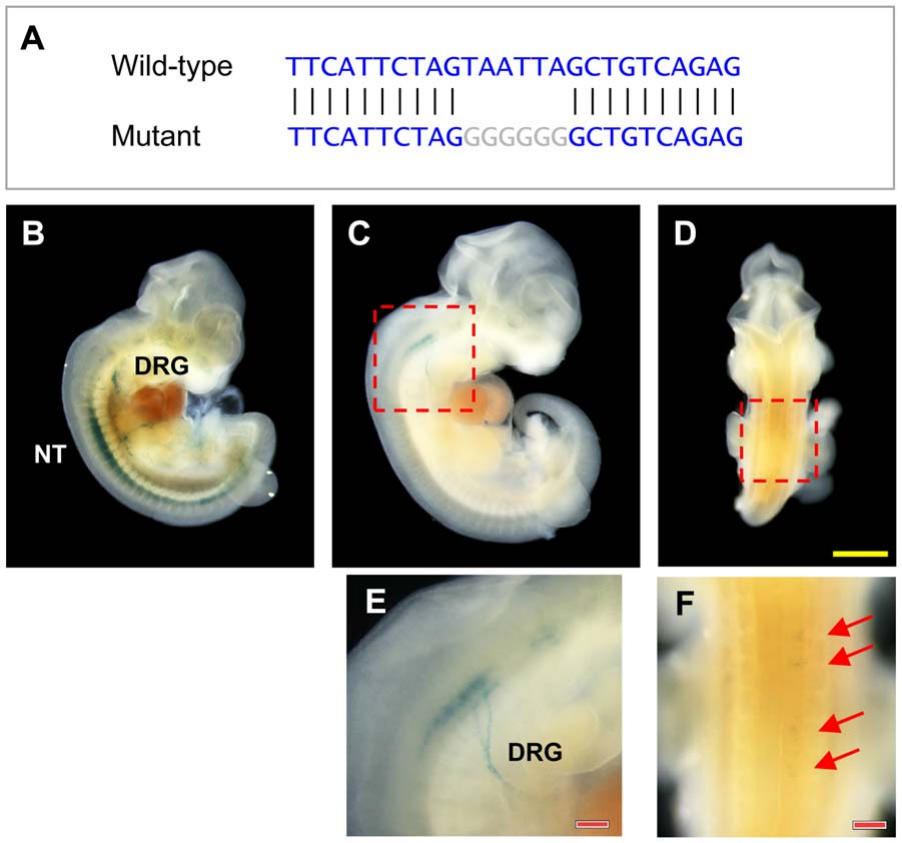
Site-directed mutagenesis of overlapping motifs in *CNE2*. (A) Wild-type and mutant sequences of overlapping motifs in *CNE2*. (B) *lacZ* expression although still detectable in neural tube and dorsal root ganglia, is greatly reduced especially in the anterior neural tube. (C, D) Faint *lacZ* expression in neural tube is present in two different embryos, and visible under higher magnification in (E, F). Yellow scale bar denotes 1 mm in length. Red scale bar denotes 0.2 mm in length.

Among the 10 CNEs predicted by us in the *Lhx2* locus, *CNE2* contained 1 instance each of motifs #2 (with 1 base mismatch) and #4 (2 mismatches) (Table 3); and *CNE4* contained 1 instance each of motifs #1 (2 mismatches) and #4 (2 mismatches). No other motif instance could be found in these or the other CNEs. The locations of these putative motif instances are shown in Figures S8, S9, S10, S11, S12, S13, S14, S15, S6, S17. Interestingly, while *CNE2* is part of the *CNE2/3* construct that directed expression to neural tube and dorsal root ganglia, *CNE4* did not drive expression of reporter gene in transgenic mouse embryos at E11.5. This suggests that *CNE4* may be active in the central nervous system at some other stage of development. Alternatively, motif #1 has occurred in this CNE by chance and is not acting as a transcription factor binding site. The latter hypothesis is more likely because the motif #1 instance is not conserved in fugu (see Figure S9). To determine if the motifs predicted in *CNE2* are functional, we selected *CNE2/3* construct, which directed expression to the neural tube and dorsal root ganglia. This construct has two motif instances ‘AGTAATTA’ and ‘GTAATTAG’ that overlap each other (bases that match motifs #4 and #2 are underlined) and are located at the 5′ end of *CNE2* (Figure S9). We mutated the ‘TAATTA’ subsequence to ‘GGGGGG’ (Figure 8A). We then tested the mutant construct in transgenic mice at stage E11.5. A total of 13 transgenic embryos were obtained. The expression in 3 embryos (23%) displayed *lacZ* expression patterns that are faintly similar to but significantly truncated compared with the expression pattern of the wild-type construct (Figure 8B–F). Two embryos (15%) showed ubiquitous *lacZ* expression throughout the entire embryo and notably, while 8 embryos (62%) showed no *lacZ* expression. Hence, through the mutation of the predicted motif, the enhancer activity of *CNE2/3* was either significantly reduced or completely abolished.

## Discussion

In this study, we have used evolutionary constraint as an indicator for identifying conserved enhancers directing the expression of *Lhx2* gene. By aligning *Lhx2* locus from distantly related vertebrates such as human and fugu, we identified 10 CNEs in the greater *Lhx2* locus that encompasses three genes. We tested the 10 CNEs as a set of eight constructs in transgenic mice and found that four elements confer restricted expression patterns in transgenic mouse embryos at E11.5. These four CNEs directed reporter gene expression to mainly the hindbrain and the neural tube, partially recapitulating the endogenous expression pattern of *Lhx2* at E11.5. In addition, one of these CNEs (*CNE10*) directed reporter expression to the rostroventral midbrain while another CNE (*CNE2/3*) induced reporter expression in the dorsal root ganglia. As for the four CNEs that did not display specific spatiotemporal reporter gene expression, they may direct expression at other stages of development or they may act as transcriptional silencers instead of enhancers, and therefore would not be detected by the minimal promoter-reporter construct used.

*Lhx2* is located within a conserved syntenic block that spans at least 14 genes in tetrapods (Figure 1). However, the conserved syntenic block covers only three genes in mammals and fish, namely *Crb2*, *Dennd1a* and *Lhx2*. Furthermore, the duplicated copy of the intervening gene *Dennd1a* has been lost in the fugu *Crb2*-*Lhx2* locus. The *Crb2-Lhx2* is reminiscent of “genomic regulatory blocks” (GRBs) previously described by Kikuta et al. (2007) based on conserved syntenic blocks of genes and CNEs in mammals and teleost fishes. GRBs are long-range conserved syntenic regions characterized by the presence of CNEs and their target genes in addition to “bystander” genes that are specifically not under the control of the CNEs. Consequently, the bystander genes can be “lost” from the GRBs in some species such as teleosts that have experienced a “fish-specific” whole-genome duplication. The loss of the duplicated *Dennd1a* in the fugu *Crb2-Lhx2* locus indicates that this could be a bystander gene in this locus. The four CNEs that were showed to be functional enhancers in our transgenic mouse assay directed expression, among other domains, to the midbrain and hindbrain. Since both *Crb2* and *Lhx2* are expressed in these domains in mouse embryos at E11.5, it is possible that these CNEs may be directing the expression of *Lhx2* and/or *Crb2*. Interestingly all four functional CNEs have one expression domain in common – the full length of the neural tube, in particular the ventral region. Since both *Lhx2* and *Crb2* are not expressed in this domain, the possibility remains that these enhancers may be directing the expression of other genes, possibly those located downstream of *Lhx2* for which we have not yet determined to be syntenic in fishes and mammals. An alternative possibility is that the Lhx2 locus may contain silencers that repress the expression of *Lhx2* and *Crb2* in the ventral region of the neural tube.

By identifying a large number of CNEs associated with TF encoding-genes that predominantly express in the central nervous system, we predicted motifs that are over-represented in such CNEs. We then hypothesized that motifs may represent TF-binding sites that are essential for directing expression to the central nervous system. We tested this hypothesis through site-directed mutagenesis of a predicted motif instance, motif #2, in *CNE2/3*, that directed expression to the neural tube. Mutation of the motif ‘TAATTA’ in this CNE resulted in the abolition of reporter gene expression in the neural tube. This indicates that the motif is necessary for the *CNE2/3* enhancer to direct gene expression to the neural tube. This motif shares the ‘TAATT’ sequence with the homeodomain binding model by Bailey et al. [53] and a forebrain-associated motif of Pennacchio et al. [29]. However in these studies, the motifs were not tested for functional activity in the central nervous system. We have now tested and provided experimental evidence that motif #2 is indeed necessary for expression in the neural tube. Interestingly, *CNE2/3* also directed expression to the dorsal root ganglia which is part of the peripheral nervous system, and mutation of motif #2 in this construct resulted in abolition of expression in this domain. These results suggest that motif #2 also plays a role driving expression in the peripheral nervous system in the embryo.

Out of the four CNEs that showed expression in the central nervous system and the dorsal root ganglia, three showed overlapping expression in the hindbrain and all showed similar expression in the neural tube. These overlapping expression patterns of the different enhancers suggest that they are either associated with different genes in this locus or alternatively, redundant enhancers of the same gene. Similar enhancers showing overlapping patterns of expression have been previously identified in the screening of mammal-chicken CNEs in the *Sox10* locus [59] and in a 1-Mb region surrounding the *Shh* locus [61]. Such apparently redundant enhancers are believed to be retained to ensure robust and high levels of expression of target genes and/or to serve as templates for evolution of novel enhancers. Interestingly, based on studies in *Drosophila*, some apparently redundant enhancers have been found to be essential for survival in extreme conditions because they maintain optimal levels of target gene expression and confer phenotypic robustness [62], [63]. For example, deletion of two enhancers that direct *shavenbaby* expression in overlapping domains in *Drosophila* results in only minor defects in trichome development under normal conditions, whereas a significant loss of trichomes is observed at extreme temperatures [62]. Such apparently redundant enhancers have been termed as “shadow” or secondary enhancers which function in concert with primary enhancers that reside closer to the target gene. It remains to be seen if the apparently redundant enhancers identified in the *Lhx2* locus act as shadow enhancers.

As the four functional CNEs that we have identified in the *Lhx2* locus do not direct expression to the forebrain, eye, limb buds and dorsal neural tube in which *Lhx2* normally expresses at E11.5, additional analyses have to be conducted to identify the complete repertoire of *cis-*regulatory elements that recapitulate the full expression pattern of *Lhx2*. It is possible that some *cis*-regulatory elements that direct specific spatiotemporal expression of *Lhx2* lie outside the locus studied. Alternatively, the *cis*-regulatory elements present within the genomic region we have analyzed are divergent in mammals and fish and cannot be detected by human-fish sequence comparisons. Comparisons among several mammalian species may help to identify such lineage-specific enhancers.

## Supporting Information

Table S1 **Primer sequences of the eight CNE constructs.**
(PDF)

Table S2 **Over-represented 8-mer motifs in CNEs of genes of clusters #1, 2, 4, 5 and 6.**
(PDF)

Figure S1 ***CNE1*** **does not act as a transcriptional enhancer at E11.5.**
(PDF)

Figure S2 ***CNE2/3*** **directs reporter gene expression in the neural tube and dorsal root ganglia at E11.5.**
(PDF)

Figure S3 ***CNE5/6*** **directs reporter gene expression in the neural tube at E11.5.**
(PDF)

Figure S4 ***CNE7*** **directs reporter gene expression in the hindbrain and neural tube at E11.5.**
(PDF)

Figure S5 ***CNE8*** **does not act as an enhancer at E11.5.**
(PDF)

Figure S6 ***CNE9*** **does not act as an enhancer at E11.5.**
(PDF)

Figure S7 ***CNE10*** **directs reporter gene expression in the midbrain, hindbrain and neural tube at E11.5.**
(PDF)

Figure S8 **Human-mouse-fugu alignment and predicted TFBS of** ***CNE1*** **.**
(PDF)

Figure S9 **Human-mouse-fugu alignment and predicted TFBS of** ***CNE2*** **.**
(PDF)

Figure S10 **Human-mouse-fugu alignment and predicted TFBS of** ***CNE3*** **.**
(PDF)

Figure S11 **Human-mouse-fugu alignment and predicted TFBS of** ***CNE4*** **.**
(PDF)

Figure S12 **Human-mouse-fugu alignment and predicted TFBS of** ***CNE5*** **.**
(PDF)

Figure S13 **Human-mouse-fugu alignment and predicted TFBS of** ***CNE6*** **.**
(PDF)

Figure S14 **Human-mouse-fugu alignment and predicted TFBS of** ***CNE7*** **.**
(PDF)

Figure S15 **Human-mouse-fugu alignment and predicted TFBS of** ***CNE8*** **.**
(PDF)

Figure S16 **Human-mouse-fugu alignment and predicted TFBS of** ***CNE9*** **.**
(PDF)

Figure S17 **Human-mouse-fugu alignment and predicted TFBS of** ***CNE10*** **.**
(PDF)
