# Supplementary material for: Mouse Transgenesis Identifies Conserved Functional Enhancers and cis-Regulatory Motif in the Vertebrate LIM Homeobox Gene Lhx2 Locus
Source: PLoS One. 2011 May 23;6(5):e20088. doi: 10.1371/journal.pone.0020088 (PMC3100342; doi:10.1371/journal.pone.0020088)
Supplement: Table S1 — Primer sequences of the eight CNE constructs. (PDF) [file pone.0020088.s001.pdf]

Table S1. Primer sequences of the eight CNE constructs.
The sequences were amplified from mouse genomic DNA by PCR. Leading attB1/attB2 sites for Gateway cloning are not shown.

CNE construct	Length of amplified sequence (bp)	Forward primer (5' to 3')	Reverse primer (5' to 3')
CNE1	279	GCT GCT CCA GGA TCG TGC TG	CAG AGC GAA GAC CCT GTG TG
CNE2/3	498	AGA CTC TAG CCC ATC TCA TTC TTC	CCA GCA TTT ACT TTG ACA TCT CTG
CNE4	397	GCT TGG AGC CAA GAG AAA ATG	CTT GCT CAG GTC TGC CTT GTA C
CNE5/6	719	CTC CGA TGT GTT GGT CCC AGC CAG	GAG CTG TCT GCA CTG TTC AGT GTG
CNE7	305	GAG TAC CAG GCA GTC AAT CAT GAG	GCT GGG AAG CCT TGT CTA ATC CTG
CNE8	382	GAC TCT GTA TTG TGG CTA CAG ATC	TGA CAT AGC CTG TGG CTG TGA TAG
CNE9	261	GTG GTC TGA CTG TGT CAC AG	CTG TCA CGG AAG TAA TGC AG
CNE10	479	GTG CTT TAC TTT TTG TCT TTG GTT CC	TAT TGA AGG CAA TTT GCA GCT GGC
