# Supplementary material for: Mouse Transgenesis Identifies Conserved Functional Enhancers and cis-Regulatory Motif in the Vertebrate LIM Homeobox Gene Lhx2 Locus
Source: PLoS One. 2011 May 23;6(5):e20088. doi: 10.1371/journal.pone.0020088 (PMC3100342; doi:10.1371/journal.pone.0020088)
Supplement: Table S2 — Over-represented 8-mer motifs in CNEs of genes of clusters #1, 2, 4, 5 and 6. (PDF) [file pone.0020088.s002.pdf]

Table S2. Over-represented 8-mer motifs in CNEs of genes of clusters #1, 2, 4, 5 and 6. Human-fugu CNEs of human transcription factor-encoding genes in each cluster were compared against CNEs of genes in other clusters. Number of instances of each motif was determined by searching each motif in the human-fugu CNEs permitting at most 2 mismatches. *P*-values were calculated based on a chi-squared test followed by Bonferroni correction for multiple testing.

**Cluster no.**	**Motif no.**	**Motif (and reverse-complement)**	**No. of instances**	***P*-value**
1	1	CGATTAAC (GTTAATCG)	110	2.68×10^{-7}
	2	CGCAATTA (TAATTGCG)	155	2.09×10^{-6}
	3	CGATTGAT (ATCAATCG)	124	2.08×10^{-5}
	4	CATTAATC (GATTAATG)	129	5.57×10^{-4}
2	1	GTTTGCGT (ACGCAAAC)	42	4.68×10^{-39}
	2	AGCAGGCG (CGCCTGCT)	21	1.02×10^{-5}
	3	TTTACACG (CGTGTAAA)	29	3.55×10^{-5}
	4	GCGGGAAA (TTTCCCGC)	21	4.09×10^{-5}
4	1	AATTAATT (AATTAATT)	120	2.55×10^{-20}
	2	GATTAATT (AATTAATC)	98	5.08×10^{-9}
5	1	CGGCTGTT (AACAGCCG)	29	6.26×10^{-10}
	2	CGCTAATG (CATTAGCG)	29	3.06×10^{-9}
	3	AATAGCCG (CGGCTATT)	24	9.75×10^{-9}
	4	GCTGTTAA (TTAACAGC)	33	1.35×10^{-7}
	5	TAAACAGC (GCTGTTTA)	29	4.90×10^{-4}
	6	TTAATTGC (GCAATTAA)	45	1.57×10^{-3}
	7	AACAGCGG (CCGCTGTT)	19	5.75×10^{-3}
	8	GCTGTTGA (TCAACAGC)	23	6.83×10^{-3}
	9	TAACAGCG (CGCTGTTA)	17	7.05×10^{-3}
	10	CGCTATTA (TAATAGCG)	18	8.18×10^{-3}
	11	GCCGTAAT (ATTACGGC)	17	8.35×10^{-3}
	12	CGTTTAAT (ATTAAACG)	60	9.18×10^{-3}
6	1	TCAATCGG (CCGATTGA)	58	1.38×10^{-6}
	2	GCCGTAAT (ATTACGGC)	62	7.50×10^{-4}
