# Supplementary material for: Mouse Transgenesis Identifies Conserved Functional Enhancers and cis-Regulatory Motif in the Vertebrate LIM Homeobox Gene Lhx2 Locus
Source: PLoS One. 2011 May 23;6(5):e20088. doi: 10.1371/journal.pone.0020088 (PMC3100342; doi:10.1371/journal.pone.0020088)

Figure S1. *CNE1* does not act as a transcriptional enhancer at E11.5.
Ventral, lateral and dorsal views of three transgenic embryos of *CNE1-pHsp68-lacZ* construct. (A) *lacZ* expresses almost ubiquitously in the dorso-posterior regions of the embryo, and only in the ventral regions of the forebrain, midbrain and hindbrain. (B) *lacZ* expression is detected in the hindbrain, dorsal root ganglia (red arrows) and neural tube. (C) Barely noticeable *lacZ* expression in dorsal root ganglia (red arrows). Scale bar denotes 1 mm in length.

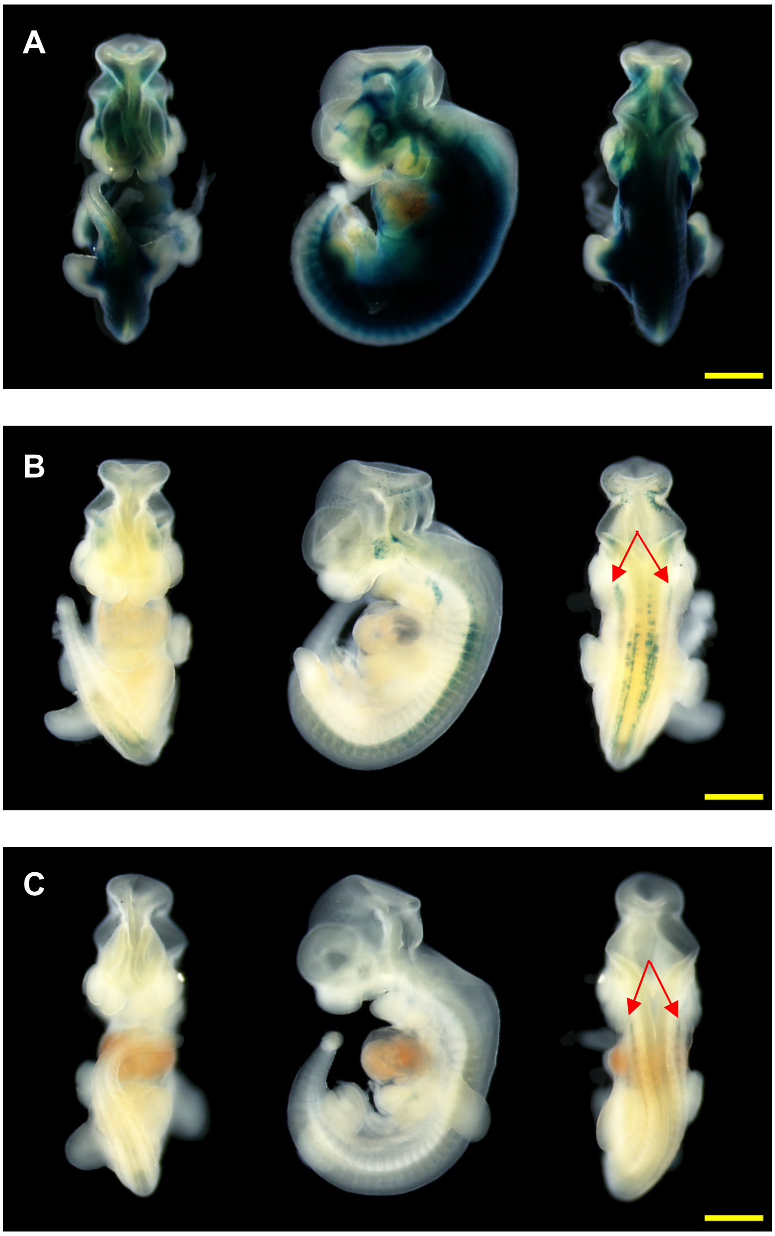

Supplement: Figure S1 — CNE1 does not act as a transcriptional enhancer at E11.5. (PDF) [file pone.0020088.s003.pdf]
