# Supplementary material for: Mouse Transgenesis Identifies Conserved Functional Enhancers and cis-Regulatory Motif in the Vertebrate LIM Homeobox Gene Lhx2 Locus
Source: PLoS One. 2011 May 23;6(5):e20088. doi: 10.1371/journal.pone.0020088 (PMC3100342; doi:10.1371/journal.pone.0020088)

Figure S2. ***CNE2/3*** **directs reporter gene expression in the neural tube and dorsal root ganglia at E11.5.**

Ventral, lateral and dorsal views of three transgenic embryos of *CNE2/3-pHsp68-lacZ* construct. (A) *lacZ* expresses strongly in the hindbrain, neural tube and dorsal root ganglia. (B) *lacZ* expression in the neural tube, dorsal root ganglia and also the dorsal medial regions of the telencephalon, diencephalon and midbrain. (C) *lacZ* expression in the neural tube and dorsal root ganglia. Scale bar denotes 1 mm in length.

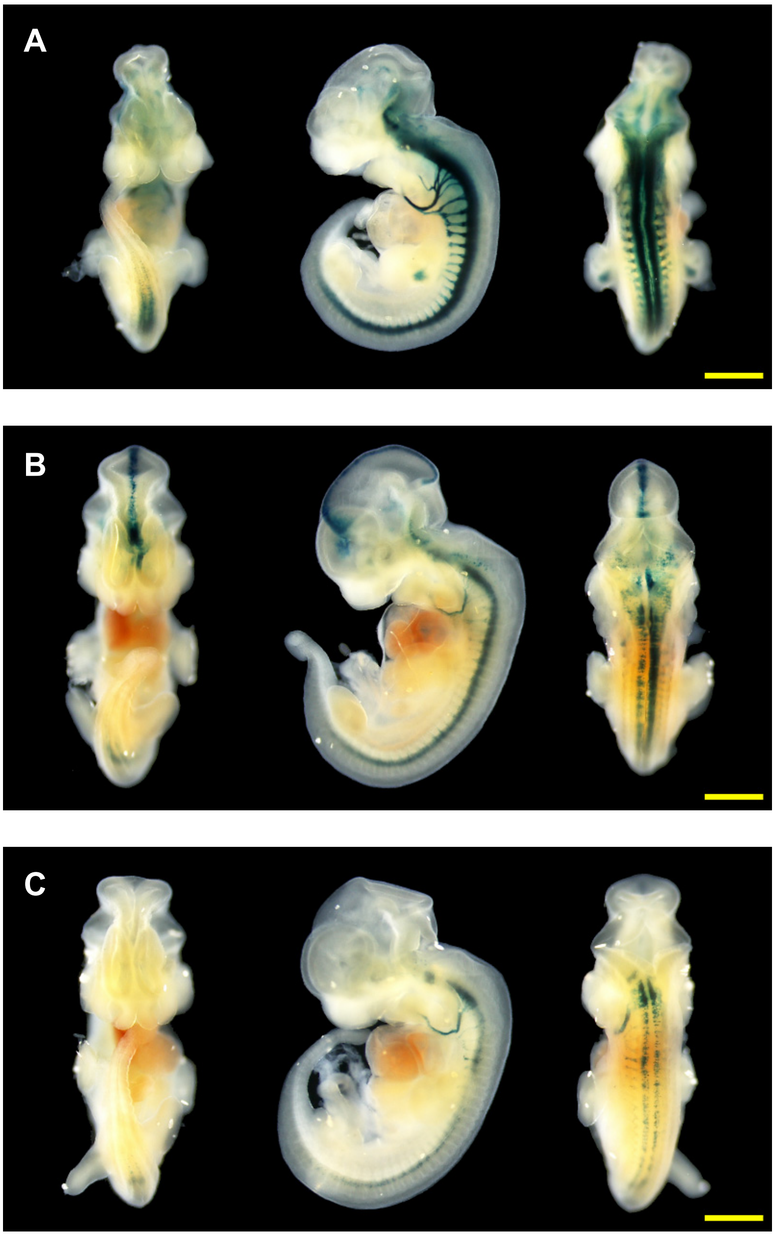

Supplement: Figure S2 — CNE2/3 directs reporter gene expression in the neural tube and dorsal root ganglia at E11.5. (PDF) [file pone.0020088.s004.pdf]
