# Supplementary material for: Mouse Transgenesis Identifies Conserved Functional Enhancers and cis-Regulatory Motif in the Vertebrate LIM Homeobox Gene Lhx2 Locus
Source: PLoS One. 2011 May 23;6(5):e20088. doi: 10.1371/journal.pone.0020088 (PMC3100342; doi:10.1371/journal.pone.0020088)
Supplement: Figure S3 — CNE5/6 directs reporter gene expression in the neural tube at E11.5. (PDF) [file pone.0020088.s005.pdf]

Figure S3. ***CNE5/6*** **directs reporter gene expression in the neural tube at E11.5.** Ventral, lateral and dorsal views of three transgenic embryos of *CNE5/6-pHsp68-lacZ* construct. (A, B) *lacZ* expression in the neural tube, extending into the ventral region of the hindbrain. (C) *lacZ* expression occurs throughout the embryo, including the ventral hindbrain and neural tube. (D) Extensive *lacZ* expression in the entire head and in the dorsal part of the embryo. Scale bar denotes 1 mm in length.

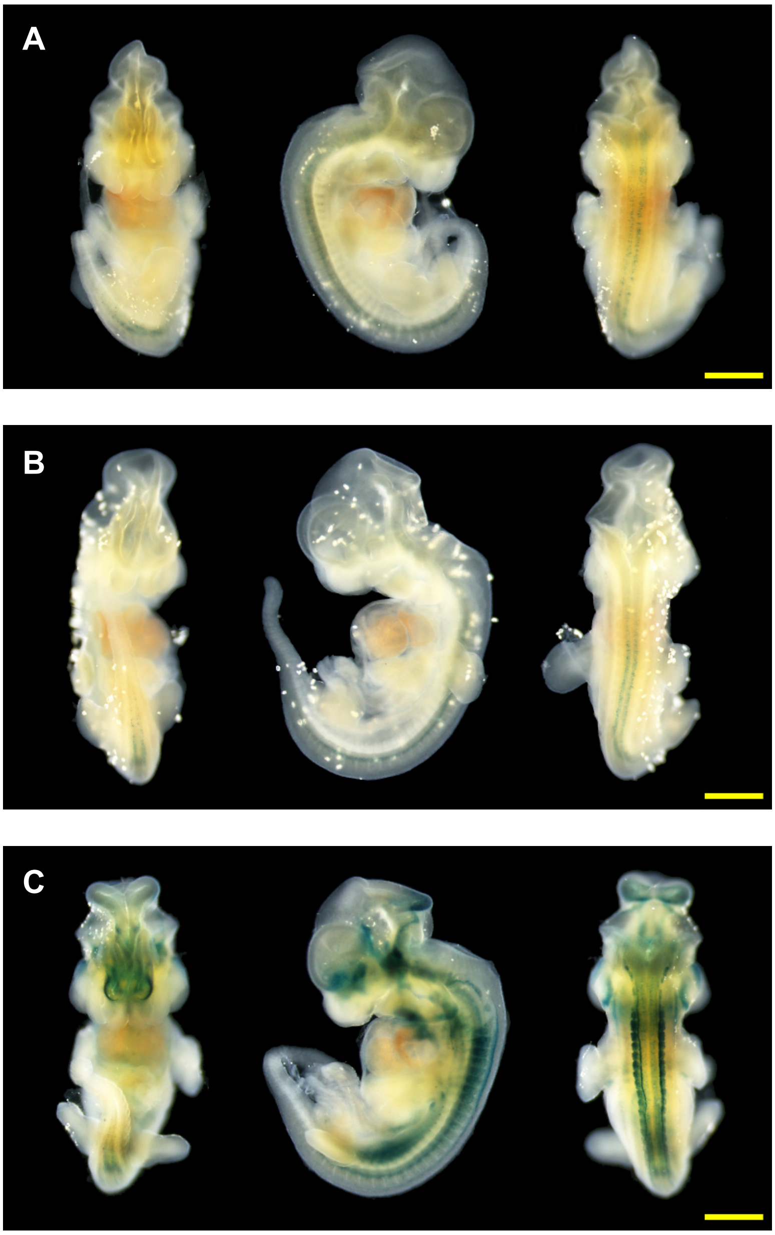

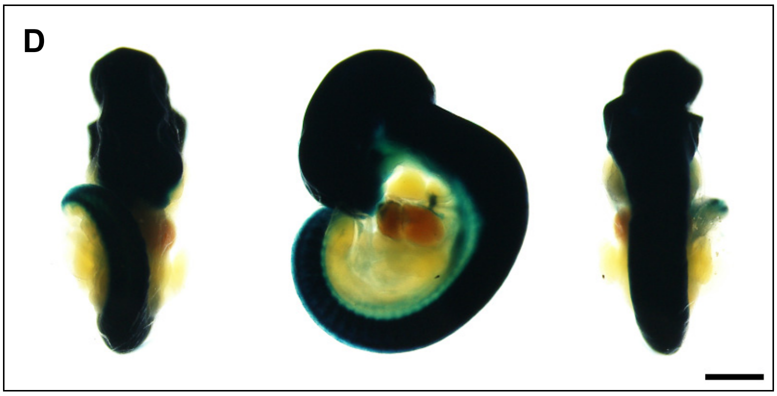
D
