# Supplementary material for: Mouse Transgenesis Identifies Conserved Functional Enhancers and cis-Regulatory Motif in the Vertebrate LIM Homeobox Gene Lhx2 Locus
Source: PLoS One. 2011 May 23;6(5):e20088. doi: 10.1371/journal.pone.0020088 (PMC3100342; doi:10.1371/journal.pone.0020088)

Figure S4. ***CNE7*** **directs reporter gene expression in the hindbrain and neural tube at E11.5.**
Ventral, lateral and dorsal views of two transgenic embryos of *CNE7-pHsp68-lacZ* construct. (A) *lacZ* expression in the hindbrain and neural tube. (B) *lacZ* expression is observed not only in the hindbrain and neural tube, but also ectopically in the liver and stomach. Scale bar denotes 1 mm in length.

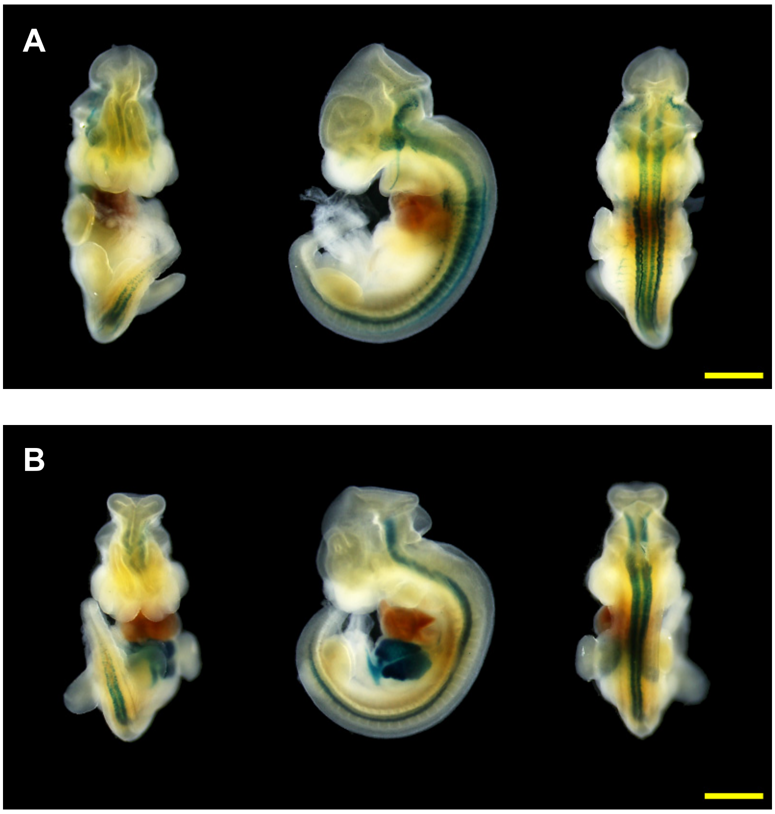

Supplement: Figure S4 — CNE7 directs reporter gene expression in the hindbrain and neural tube at E11.5. (PDF) [file pone.0020088.s006.pdf]
