# Supplementary material for: Mouse Transgenesis Identifies Conserved Functional Enhancers and cis-Regulatory Motif in the Vertebrate LIM Homeobox Gene Lhx2 Locus
Source: PLoS One. 2011 May 23;6(5):e20088. doi: 10.1371/journal.pone.0020088 (PMC3100342; doi:10.1371/journal.pone.0020088)

Figure S5. ***CNE8*** **does not act as an enhancer at E11.5.**
Ventral, lateral and dorsal views of two transgenic embryos of *CNE8-pHsp68-lacZ* construct. (A, B) Both embryos exhibited ectopic *lacZ* expression in various anatomical structures with no reproducible similarities. Scale bar denotes 1 mm in length.

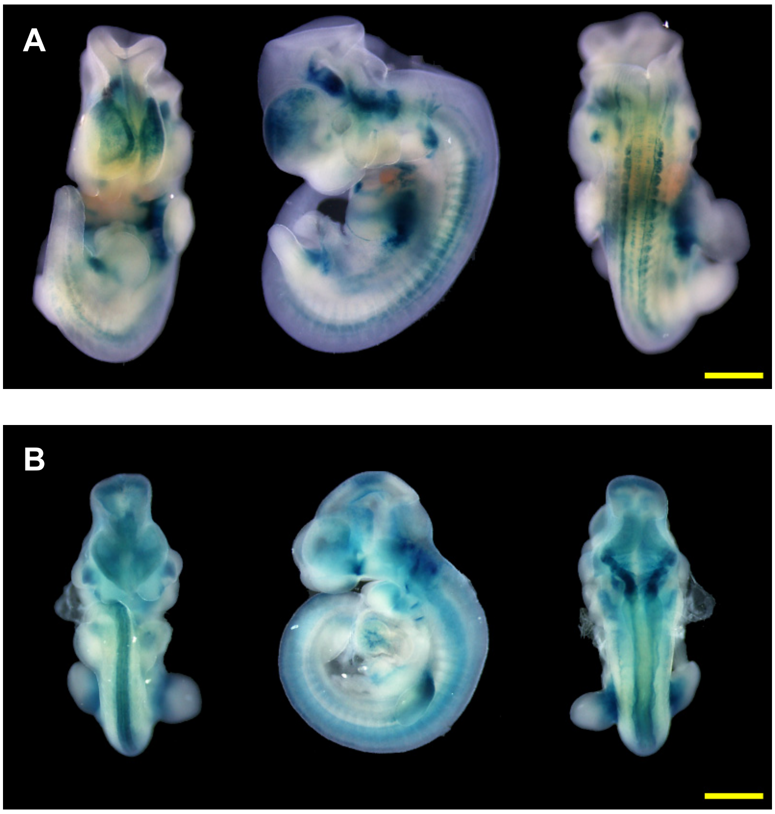

Supplement: Figure S5 — CNE8 does not act as an enhancer at E11.5. (PDF) [file pone.0020088.s007.pdf]
