# Supplementary material for: Mouse Transgenesis Identifies Conserved Functional Enhancers and cis-Regulatory Motif in the Vertebrate LIM Homeobox Gene Lhx2 Locus
Source: PLoS One. 2011 May 23;6(5):e20088. doi: 10.1371/journal.pone.0020088 (PMC3100342; doi:10.1371/journal.pone.0020088)
Supplement: Figure S6 — CNE9 does not act as an enhancer at E11.5. (PDF) [file pone.0020088.s008.pdf]

Figure S6. *CNE9* does not act as an enhancer at E11.5.
Ventral, lateral and dorsal views of all four transgenic embryos of *CNE9-pHsp68-lacZ* construct that exhibit *lacZ* expression. (A – D) All embryos exhibited ectopic *lacZ* expression in various anatomical structures with no reproducible similarities. Scale bar denotes 1 mm in length.

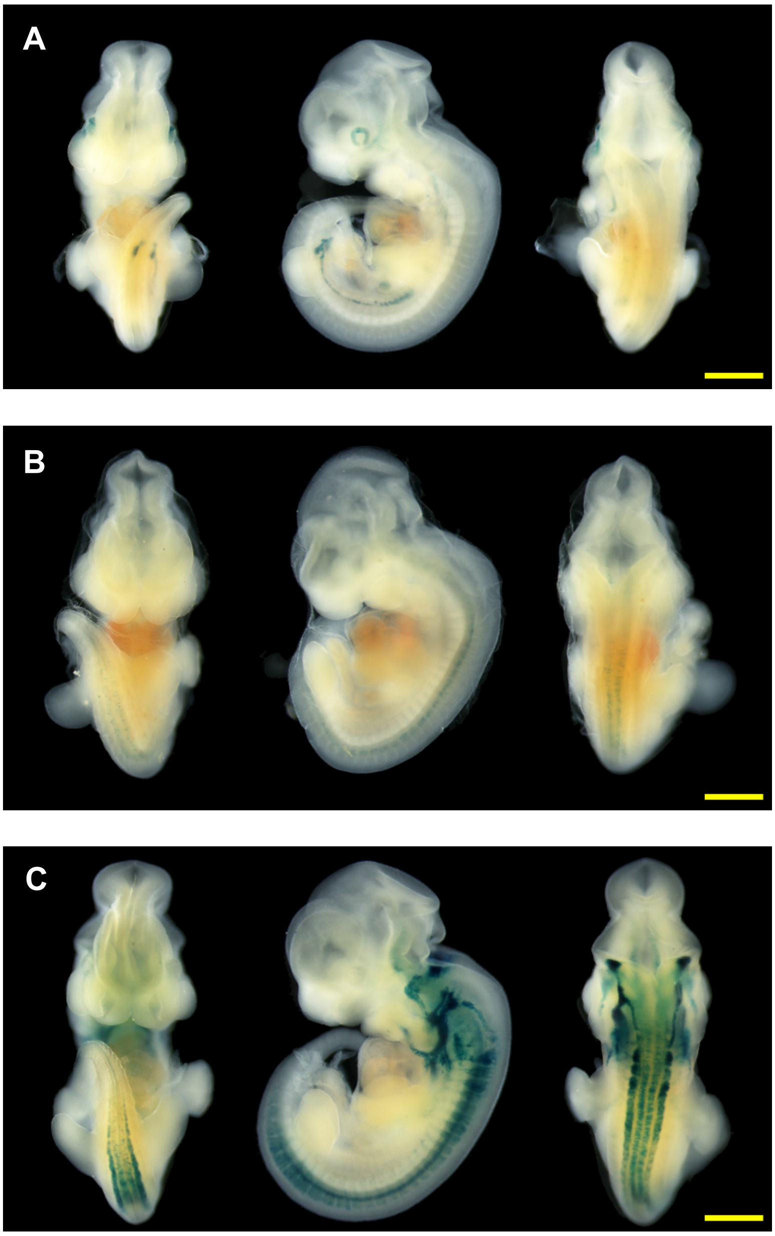

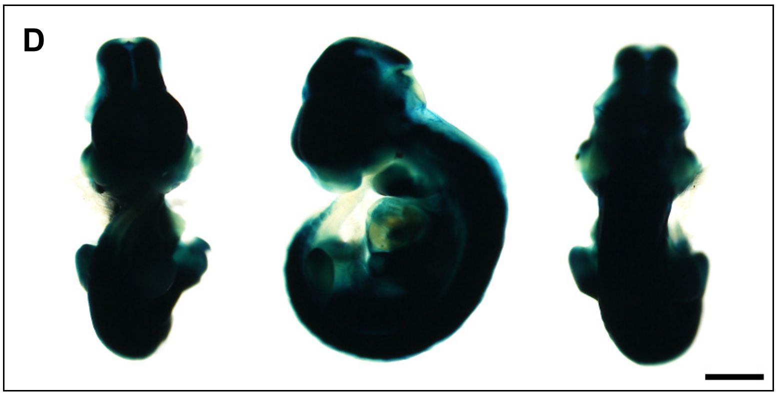
D
