# Supplementary material for: Mouse Transgenesis Identifies Conserved Functional Enhancers and cis-Regulatory Motif in the Vertebrate LIM Homeobox Gene Lhx2 Locus
Source: PLoS One. 2011 May 23;6(5):e20088. doi: 10.1371/journal.pone.0020088 (PMC3100342; doi:10.1371/journal.pone.0020088)

Figure S7. *CNE10* directs reporter gene expression in the midbrain, hindbrain and neural tube at E11.5.
Ventral, lateral and dorsal views of two transgenic embryos of *CNE10-pHsp68-lacZ* construct that exhibit *lacZ* expression. (A, B) *lacZ* expression was observed in the midbrain, hindbrain and neural tube for both embryos with additional ectopic expression in the eye for (A) and additional expression in the heart for (B). Scale bar denotes 1 mm in length.

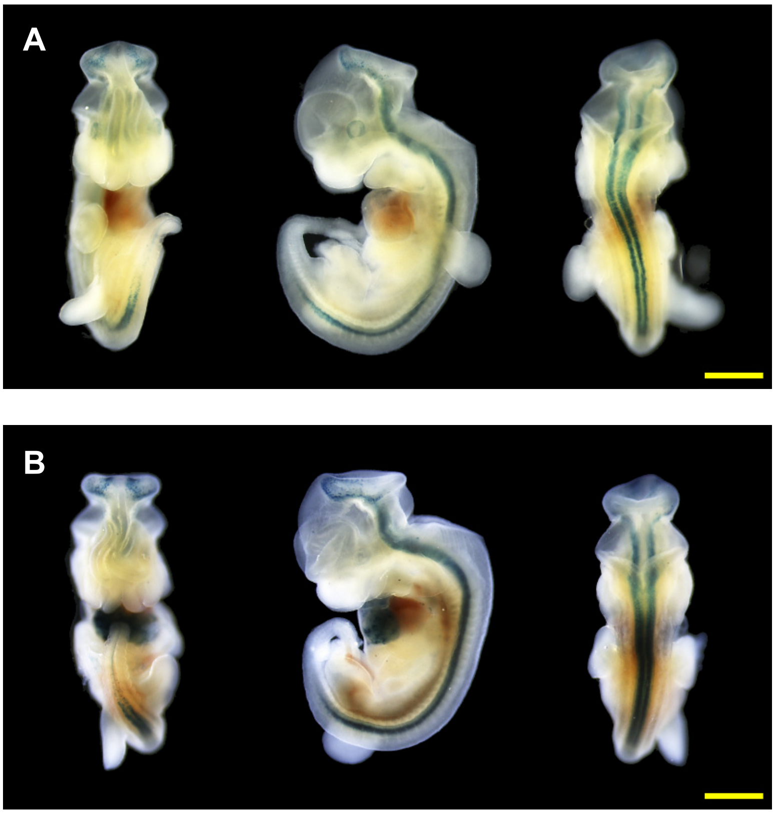

Supplement: Figure S7 — CNE10 directs reporter gene expression in the midbrain, hindbrain and neural tube at E11.5. (PDF) [file pone.0020088.s009.pdf]
