# Supplementary material for: Mouse Transgenesis Identifies Conserved Functional Enhancers and cis-Regulatory Motif in the Vertebrate LIM Homeobox Gene Lhx2 Locus
Source: PLoS One. 2011 May 23;6(5):e20088. doi: 10.1371/journal.pone.0020088 (PMC3100342; doi:10.1371/journal.pone.0020088)
Supplement: Figure S8 — Human-mouse-fugu alignment and predicted TFBS of CNE1 . (PDF) [file pone.0020088.s010.pdf]

Figure S8. Human-mouse-fugu alignment and predicted TFBS of *CNE1*.
TFBS on the forward strand are shown in blue and TFBS on the reverse strand are shown in red below the human-mouse-fugu sequence alignment. The binding TF and position weight matrix similarity score are listed next to each predicted site.

```
human    AGCACGTCACGCTGGCCC-----AAAATGAAATGCTGACAAATGTAGATATTTCAAATTT 55
mouse    CGCACGTCACGCTGGCCC-----GAAATGAAGTGCTGACAAATGTAGATGTTTCAAATTT 55
fugu     AGCACCTTATAATGGGCCCAGCACAAATGGTATGTTGACAAATGCAGATATTTCAATTTT 60
                                 aatgaaatGCTGacaaatgta 0.993  MARE.01
                                                       atattTCAAattt 0.956  CHR.01
                                                          tttcaaattT... 0.999  S8.01
                                                           ttcaaattT... 0.936  LHX3.02
                                                           ttcaaatTT... 0.93  HMX3.02
                                                              aaatTT... 0.93  HMX3.02
                                                               aattT... 0.956  GSH2.01
                                                               aattT... 0.961  BRIGHT.01
                                                                 tTT... 0.941  NKX61.02


human    AATTGACATTTAAATGTACTT                                        76
mouse    AATTGACATTTAAATGCACTT                                        76
fugu     AATTGACACCACGAGGCAGTT                                        81
      ...AATtgac 0.999  S8.01
      ...AATtgaca 0.936  LHX3.02
      ...AAttga 0.93  HMX3.02
      ...AAttgacat 0.93  HMX3.02
      ...AATtgacattta 0.956  GSH2.01
      ...AATtgaca 0.961  BRIGHT.01
      ...AAttgaca 0.941  NKX61.02
         aatTGACattt 1  TGIF.01
           ttgacatTTAAatgt 0.949  HMX3.02
              acatTTAAatgtact 0.949  HMX3.02
```
