# Supplementary material for: Mouse Transgenesis Identifies Conserved Functional Enhancers and cis-Regulatory Motif in the Vertebrate LIM Homeobox Gene Lhx2 Locus
Source: PLoS One. 2011 May 23;6(5):e20088. doi: 10.1371/journal.pone.0020088 (PMC3100342; doi:10.1371/journal.pone.0020088)
Supplement: Figure S9 — Human-mouse-fugu alignment and predicted TFBS of CNE2 . (PDF) [file pone.0020088.s011.pdf]

Figure S9. Human-mouse-fugu alignment and predicted TFBS of *CNE2.*
TFBS on the forward strand are shown in blue and TFBS on the reverse strand are shown in red below the human-mouse-fugu sequence alignment. The binding TF and position weight matrix similarity score are listed next to each predicted site. Sequences enclosed in blue (forward strand) or red (reverse strand) boxes are instances of motifs over-represented in the CNEs of central nervous system-expressed TF-encoding genes. Motif numbers are listed and described in Table 3.

```
                                 Motif #4       Motif #2
human    ATTGTTCCTTAGCGGTATACATGCTTCATTCTAGTAATTAGCTGTCAGAGAGGACCCGAC 60
mouse    ATTGTTCCTGAGTGGTATACACACTTCATTCTAGTAATTAGCTGTCAGAGAGGACCCGAC 60
fugu     ATTGTTCTCTATCACTGGACAAGGCTGATTCCTGTAATTAGCTGTCAGCACAGAGCCCTC 60
                                  tcattctagTAATtagc 0.997  S8.01
                                    attctagTAATtagctgtc 0.925  BRN3.02
                                    attctagtaATTAgctg 0.968  GSH2.01
                                       ctagTAATtagctgtca 0.958  GSH2.01
                                         agtaATTAgctgtcaga 0.992  S8.01
                                                agctGTCAgag 1  TGIF.01

human    AGCAGCTTAATGTTGTCCGCTCAAATCATATTAATGAAATGCAATCCACTCAAATGTCCT 120
mouse    AGCAGCCTAATGTTGTCCGCTCCAATCATATTAATGAAATGCAATCCACTCAGATGTCCT 120
fugu     AGCAGCTTAGTGCTGTCCGCTCAAATCAAACTAATGAAATGCAATCCACTCAAATGTCCT 120
                             tcaAATCatattaat 0.901  GFI1.02
                             tcaaatcaTATTaat 0.941  SATB1.01
                                             aaatGCAAtccac 0.942  CHOP.01
                                                   aatcCACTcaaatgt 0.97  NKX32.01

human    GAATGCATATCTGTAA-----TTGCGTTAAGCAGAGAGG                      154
mouse    GAATGCATATCTGTAA-----TTGCATTAAGCAGAGAGA                      154
fugu     GGATGCATATCTGTAACAGGCCTGCACACACGAGAGCGG                      159
           atgcaTATCtgta 0.943  GATA.01
             gcatatctgTAA-----Ttgcg 0.994  S8.01
```
