# Supplementary material for: Mouse Transgenesis Identifies Conserved Functional Enhancers and cis-Regulatory Motif in the Vertebrate LIM Homeobox Gene Lhx2 Locus
Source: PLoS One. 2011 May 23;6(5):e20088. doi: 10.1371/journal.pone.0020088 (PMC3100342; doi:10.1371/journal.pone.0020088)
Supplement: Figure S10 — Human-mouse-fugu alignment and predicted TFBS of CNE3 . (PDF) [file pone.0020088.s012.pdf]

Figure S10. Human-mouse-fugu alignment and predicted TFBS of *CNE3*.
TFBS on the forward strand are shown in blue and TFBS on the reverse strand are shown in red below the human-mouse-fugu sequence alignment. The binding TF and position weight matrix similarity score are listed next to each predicted site.

```
human    ATTACGAT-------CAAGCACATTAATCCATTATTTCTTC-AGAATCTGACAGCCAATT 52
mouse    GTTACGAT-------CAAGCACATTAATCCATTACTTCTTC-AGAATCTGACAGCCAATT 52
fugu     GTTACCATTCAAATGCATGCTCATTAATCCATTAATTCATCTTGTTTTTGACAGCCAATT 60
                            cacatTAATccat 0.913  TST1.01
                             acatTAATccattattt 0.982  OTX2.01
                              cattaatccATTAtttc 0.959  BARX2.01
                                                    atcTGACagcc 1  TGIF.01
                                                           agccaaTT... 0.926  NKX61.01

human    AATA                                                         56
mouse    AATA                                                         56
fugu     AATA                                                         64
      ...AAt 0.926  NKX61.01
```
