# Supplementary material for: Mouse Transgenesis Identifies Conserved Functional Enhancers and cis-Regulatory Motif in the Vertebrate LIM Homeobox Gene Lhx2 Locus
Source: PLoS One. 2011 May 23;6(5):e20088. doi: 10.1371/journal.pone.0020088 (PMC3100342; doi:10.1371/journal.pone.0020088)
Supplement: Figure S11 — Human-mouse-fugu alignment and predicted TFBS of CNE4 . (PDF) [file pone.0020088.s013.pdf]

Figure S11. Human-mouse-fugu alignment and predicted TFBS of *CNE4.*
TFBS on the forward strand are shown in blue and TFBS on the reverse strand are shown in red below the human-mouse-fugu sequence alignment. The binding TF and position weight matrix similarity score are listed next to each predicted site. Sequences enclosed in blue boxes are instances of motifs over-represented in the CNEs of central nervous system-expressed TF-encoding genes. Motif numbers are listed and described in Table 3.

```
human    GATTTATGACTTATGACTTGTTCATATTTAATTTGTAACCAAACACTTCCCCTTCCTCTT 60
mouse    GATTTATGACTTATGACTTGTTCATATTTAATTTGTAACCAAACACTTCCCCTTCCTCTT 60
fugu     GATTTATGGCTTATGACAAACATATAT----------ACCAAACACTTCCCATTCCTCTC 50
                            gttcatattTAATttgt 0.993  MSX.01
                             ttcatattTAATttgta 0.987  LHX3.02
                                 tattTAATttgtaacca 0.963  GSH2.01
                                 tattTAATttgta 0.96  BRIGHT.01
                                   tTTAAtttgta 0.92  NKX61.01
                                              accaaacacTTCCccttcctc 0.997  SPI1_PU1.02
                                                    cacttccccTTCCtctt... 0.962  SPI1_PU1.02
                                                    cacttCCCCtt 1  MZF1.02
                 Motif #4     Motif #1

human    AGCTACCTTAGAACTCTTGATTAACTCTTGTTTCCACAGGTCAGAGGTTAAATAAAGGA  119
mouse    AGCTACCTTAGAACTCTTGATTAACTCTTGTTTTCACAGGTCAGAGGTTAAATAAAGGA  119
fugu     AACTTCCTCAGAACTCTGGAAGATGCCTTATTTTCTCAGGTCAGAGGTTAAACAATGAA  109
      ...agct 0.962  SPI1_PU1.02
                    aactcttGATTaactct 0.906  OC2.01
                            attaactcttgtTTCCaca 0.979  NFAT.01
                                      gtttccacaggtcagAGGTtaaata 0.905  RAR_RXR.01
                                        ttccacAGGTcagaggttaaataaa 0.917  TR4.02
                                              agGTCAgaggt 0.953  DREAM.01
```
