# Supplementary material for: Mouse Transgenesis Identifies Conserved Functional Enhancers and cis-Regulatory Motif in the Vertebrate LIM Homeobox Gene Lhx2 Locus
Source: PLoS One. 2011 May 23;6(5):e20088. doi: 10.1371/journal.pone.0020088 (PMC3100342; doi:10.1371/journal.pone.0020088)
Supplement: Figure S12 — Human-mouse-fugu alignment and predicted TFBS of CNE5 . (PDF) [file pone.0020088.s014.pdf]

Figure S12. Human-mouse-fugu alignment and predicted TFBS of *CNE5*.
TFBS on the forward strand are shown in blue and TFBS on the reverse strand are shown in red below the human-mouse-fugu sequence alignment. The binding TF and position weight matrix similarity score are listed next to each predicted site.

```
human    TGTCCCCTCGGCCTCACAGCTCTGCCAGTTGGCAAACCCAAGCCAGGAAGTTGTCAATTC 60
mouse    TGTCCCCTCAGCCTTCCAGCACTGCCAGTTGGCAAACCCAAGCCAGGAAGTTGTCAATTC 60
fugu     TGTCCTCTCTGCACCACTACTCTGCCAGTTGGCAAGGATATACCAGGAAACTGTCAGTGT 60
         tgtccCCTCggcc 0.919  MAZ.01
                             tctgccaGTTGgc 0.959  CMYB.01
                                              ccaagccAGGAagttgtcaat 0.958  PEA3.01
                                                         agttGTCAatt 1  TGIF.01

human    CAATTGCGTTTAGGCCATGTAGTCCTATTAAAGAAGGCCTGCTGGTT-AAGGGGCGTCCT 119
mouse    CAATTGCGTTTAGGCCATGTAGTCCTATTAAAGAAGGCTTGCTGGTT-AAGGGGCATCCT 119
fugu     CTATTGAGTTTCGTAGAGGAACTCCTGCTAAAGCAGCCTTGCTGGTTCCAGGACCCAATT 120
                               tcctATTAaagaaggcc 0.994  HHEX.01
                                                          agggGCGTcct 0.953  WHN.01
                                                                  cct... 0.991  AREB6.04

human    CTGTTTCCAAAGCATGGG                                           137
mouse    CTGTTTCCAAAGCATGGG                                           137
fugu     CTGTTTCCAAAGCATGGG                                           138
      ...ctGTTTccaa 0.991  AREB6.04
```
