# Supplementary material for: Mouse Transgenesis Identifies Conserved Functional Enhancers and cis-Regulatory Motif in the Vertebrate LIM Homeobox Gene Lhx2 Locus
Source: PLoS One. 2011 May 23;6(5):e20088. doi: 10.1371/journal.pone.0020088 (PMC3100342; doi:10.1371/journal.pone.0020088)
Supplement: Figure S13 — Human-mouse-fugu alignment and predicted TFBS of CNE6 . (PDF) [file pone.0020088.s015.pdf]

Figure S13. Human-mouse-fugu alignment and predicted TFBS of *CNE6*.
TFBS on the forward strand are shown in blue and TFBS on the reverse strand are shown in red below the human-mouse-fugu sequence alignment. The binding TF and position weight matrix similarity score are listed next to each predicted site. Sequence enclosed in blue box is an instance of a motif over-represented in the CNEs of central nervous system-expressed TF-encoding genes. Motif numbers are listed and described in Table 3.

```
human    CAACTT--TCACTGACTAGAGTTTTCAAGGAGCTATGAATGAGTTTAACAAAGGAGGTCT 58
mouse    CAACTT--CCACTGACTAGAATTTTCAAGGGGCTGTGAATGAGTTTAACAAAGGAGGTCT 58
fugu     CAGCTTTCTCCCTACTCACAGTTCACAGAGAGTCCTGAATGAGATTAACAAAAGCGTTCT 60
                                    aggagctatgAATGagt 0.919  HMGA.01
                                      gagctatgAATGagttt 0.935  HBP1.01
                                         ctatgAATGagtttaac 0.967  NANOG.01
                                                  agtttaaCAAAggaggt 0.936  LEF1.01
                                                       aacAAAGgaggtc 0.966  GKLF.02


human    GT-GGCTAGTTTCTGGCTGAAGTCCATGTCAACAGTTTTTAAGGTCACAGGCCACACTAA 117
mouse    GT-GGCTAGTTTCTGGCTGAAGTCCATGTCAACAGTTTTTAAGGTCACAGGCCACACTAA 117
fugu     GTCAGACCCCTCTTGGCCAGGTTCCACGTCAACAGCTCACAGGGTCAAGGGTCACACTAA 120
                                          agtttttaaGGTCacaggccaca 0.96  VERBA.01
                                          agtttttAAGGtcacaggc 0.916  ERR.01
                                             ttttAAGGtcacagg 0.927  NBRE.01


human    CTAACATTTATTCCCAGCAGACCC--TGTTCTAAACACAAGCTGTAAGTAGTACAAATTT 175
mouse    CTAACATTTATTCCCAGCAGACCC--TGTTCTAAACACAAGCTGTACATAGTACAAATTT 175
fugu     CT-ACATTCATTCCCAGCAGACCCTTTGGTCCAAACACAGGCTGCCAGTCACACAAACTC 179
          taACATttatt 0.979  SMARCA3.01
                      ccagcagaccc--TGTTctaa 0.907  PRE.01
                                                                    t... 0.922  MTBF.01
                                                              Motif #1
human    GGAATTTTT-CTGTAATCCCTTGAGATATTCCTGATCCTTCCACATGTTTCTGCTCAATT 234
mouse    GAAATTTTT-CTGTAATCCCTTGAGATATTCCTGATCCCTCCACATGTTTCTGCTCAATT 234
fugu     TCAGTTTTTCCTCTAATCCCTTGAGATATGTCTGATCTCTCCACATGTTTCTGCTCAATT 239
      ...ggaATTTt 0.922  MTBF.01
                 t-ctgTAATcccttgaga 0.982  GSC.01
                                      tcctgatcCTTCcacatgtttctgc 0.922  HSF2.01
                                              cttCCACatgtttctgctc 0.903  PAX6.04
                                                  cacatGTTTctgc 0.986  AREB6.04
                                                         ttctgctcaATT... 0.972  BARX2.01
                                                            tgctcaaTT... 0.923  HMX3.02
                                                             gctcaATT... 0.957  BRIGHT.01
                                                             gctcaaTT... 0.926  NKX61.01
                                                               tcaaTT... 0.92  HMX3.02


human    AAAAACAAT                                                    243
mouse    AAAAACAAT                                                    243
fugu     -AAAGCAAT                                                    247
      ...Aaaaa 0.972  BARX2.01
      ...AAaaac 0.923  HMX3.02
      ...Aaaaa 0.957  BRIGHT.01
      ...AAa 0.926  NKX61.01
      ...AAaaacaat 0.92  HMX3.02
```
