# Supplementary material for: Mouse Transgenesis Identifies Conserved Functional Enhancers and cis-Regulatory Motif in the Vertebrate LIM Homeobox Gene Lhx2 Locus
Source: PLoS One. 2011 May 23;6(5):e20088. doi: 10.1371/journal.pone.0020088 (PMC3100342; doi:10.1371/journal.pone.0020088)
Supplement: Figure S14 — Human-mouse-fugu alignment and predicted TFBS of CNE7 . (PDF) [file pone.0020088.s016.pdf]

Figure S14. Human-mouse-fugu alignment and predicted TFBS of *CNE7*.
TFBS on the forward strand are shown in blue and TFBS on the reverse strand are shown in red below the human-mouse-fugu sequence alignment. The binding TF and position weight matrix similarity score are listed next to each predicted site.

```
human    TAACACCCCTCCAGGGAACATCTCCAGCCTGACCATATGGATCAGAGTGAA          51
mouse    TAACACCCCTCCAGGGACCATCTCCAGCCTGACCATATGGATCAGAGTGAA          51
fugu     TAACAGGCCCCCGGGGACCCAGCACTGCAGGACCATATGGGGAGCAGTGAA          51
         taacacccctccaGGGAacatct 0.925  OLF1.02
                  tccagGGAAcatc 0.901  IK3.01
                               tccagcCTGAcca 0.918  MEF3.01
                                      tgaCCATatggat 0.952  NEUROG.01
                                       gaCCATatgga 0.963  SMARCA3.01
                                        accatATGGat 0.967  SMARCA3.01
```
