# Supplementary material for: Mouse Transgenesis Identifies Conserved Functional Enhancers and cis-Regulatory Motif in the Vertebrate LIM Homeobox Gene Lhx2 Locus
Source: PLoS One. 2011 May 23;6(5):e20088. doi: 10.1371/journal.pone.0020088 (PMC3100342; doi:10.1371/journal.pone.0020088)
Supplement: Figure S15 — Human-mouse-fugu alignment and predicted TFBS of CNE8 . (PDF) [file pone.0020088.s017.pdf]

Figure S15. Human-mouse-fugu alignment and predicted TFBS of *CNE8.*
TFBS on the forward strand are shown in blue and TFBS on the reverse strand are shown in red below the human-mouse-fugu sequence alignment. The binding TF and position weight matrix similarity score are listed next to each predicted site.

```
human    CCTTCATTTCTGTATAGCTGTGTTTGCCTCAGGGCCACGCTGCTTGTTTTGACAGTGGAT 60
mouse    CCTTCATTTCTGTATGGCTGTGTTTGCCTCAGGGCCACGCTGCTTGTTTTGACAGTGGAT 60
fugu     CTCTCACTGTTCAGTGAATCGGCTTGCCTTAGGGCCCTGCTGCTTGTTTTGACAGAGCTT 60
                               tttGCCTcagggcca 0.961  AP2.02
                                        gggCCACgctgct 0.959  ATF6.01
                                                       tttTGACagtg 1  TGIF.01

human    CCCCCTAGATAGTTGGGATTAGTCGAGCTTGTTTAGATAAACTTGAA-------ATCAAG 113
mouse    CCCCCTAGATAGTTGGGATTAGTCGAGCTTGTTTGGATAAACTTGAA-------ATCAAG 113
fugu     CCCCT--GATAGTTAGGATTAGTCCAGCTTGTTTGGCTAAACTAGGAGAGGGGGATCTTA 118
               agatAGTTgggat 0.944  MYT1L.01
                 atagttgggaTTAGtcg 0.969  PTX1.01
                   agttGGGAttagt 0.98  IK2.01
                      tggGATTagtc 1  TFII-IR4.01
                                 gagcttgTTTAgataaa 0.927  HFH1.01
                                        tttaGATAaactt 0.95  GATA1.03
                                                    tgaA-------ATCaag... 0.963  GFI1.01

human    GCCTTCT                                                      120
mouse    GCCTTCT                                                      120
fugu     GGCTACT                                                      125
      ...gcctt 0.963  GFI1.01
```
