# Supplementary material for: Mouse Transgenesis Identifies Conserved Functional Enhancers and cis-Regulatory Motif in the Vertebrate LIM Homeobox Gene Lhx2 Locus
Source: PLoS One. 2011 May 23;6(5):e20088. doi: 10.1371/journal.pone.0020088 (PMC3100342; doi:10.1371/journal.pone.0020088)
Supplement: Figure S16 — Human-mouse-fugu alignment and predicted TFBS of CNE9 . (PDF) [file pone.0020088.s018.pdf]

Figure S16. Human-mouse-fugu alignment and predicted TFBS of *CNE9*.
TFBS on the forward strand are shown in blue and TFBS on the reverse strand are shown in red below the human-mouse-fugu sequence alignment. The binding TF and position weight matrix similarity score are listed next to each predicted site.

```
human    CTGCTCATTACTTCTTCCTTCTGTATCGAGACCCTTATTTACATTTCAGATTA        53
mouse    CTGCTCATTACTTCTTCTTTCTGTATCAAGACCCTTATTTACATTTCAGATTA        53
fugu     CTGCTCATTAGTCTATCGCTTTGGATCAAACGCTGTATTTACATATCAAATTA        53
                                      acccttaTTTAcatttc 0.948  XFD1.01
```
