# Supplementary material for: Mouse Transgenesis Identifies Conserved Functional Enhancers and cis-Regulatory Motif in the Vertebrate LIM Homeobox Gene Lhx2 Locus
Source: PLoS One. 2011 May 23;6(5):e20088. doi: 10.1371/journal.pone.0020088 (PMC3100342; doi:10.1371/journal.pone.0020088)
Supplement: Figure S17 — Human-mouse-fugu alignment and predicted TFBS of CNE10 . (PDF) [file pone.0020088.s019.pdf]

Figure S17. Human-mouse-fugu alignment and predicted TFBS of *CNE10*.
TFBS on the forward strand are shown in blue and TFBS on the reverse strand are shown in red below the human-mouse-fugu sequence alignment. The binding TF and position weight matrix similarity score are listed next to each predicted site.

```
human    CTGTAAATAATTGACAGATAATATTAATTGGCTTTGGATTAGTGAGCAAGCAGAGGATCA 60
mouse    CTGTAAATAATTGACAGATAATATTAATTGGCTTTGGATTAGTGAGCAAGCAGAGGATCA 60
fugu     CTGTAAATAATTGACAGATAATATTAATTGGCTTTGGATTAGTGAGCAAGCTCAGTATCA 60
            taaaTAATtgacagata 0.92  PCE1.01
                 aatTGACagat 1  TGIF.01
                     gacaGATAatatt 0.98  GATA2.02
                        agataatattaATTGgctttgga 0.94  CDP.02
                        agataatatTAATtggc 0.999  S8.01
                         gataataTTAAttgg 0.929  HMX3.02
                            aataTTAAttggctt 0.935  HMX3.02
                             atatTAATtggctttgg 0.977  GSH2.01
                             atattaaTTGGcttt 0.947  CAAT.01
                             atatTAATtggct 0.949  BRIGHT.01
                               aTTAAttggct 0.926  NKX61.01
                                     tggctttggATTAgtga 0.965  CRX.01
                                                               ggatca... 0.951  NANOG.01

human    CACATTAATATTTGATCAGAAGATGATAGCAGCCCTGGCAGGGGATCCAAGGGGGGCAGC 120
mouse    CACATTAATATTTGATCAGAAGATGATAGCAGCCCTGGCAGGGGATCCAAGGGGGGCAGC 120
fugu     CACATTAATATTTGATCAGAGGATGATGGCGACCCTGGCAGGGGATCCGAAAGGGGCAGC 120
      ...caCATTaatat 0.951  NANOG.01
            attAATAtttgatca 0.948  SATB1.01
                       atcagAAGAtgatagca 0.97  EVI1.07
                                            tggcaGGGGatcc 0.945  INSM1.01
                                                           ggggGGCAgc... 0.92  MYOD.01
                                                            gggggcaGC... 0.908  MYOGENIN.02

human    TGAGGCTGCTATGTTGATGAGCACA                                     145
mouse    TGAGGCTGCTATGTTGATGAGCACA                                     145
fugu     TGAGGCTGTTTTGTTGATTAGGCCA                                     145
      ...tgaggct 0.92  MYOD.01
      ...TGaggctg 0.908  MYOGENIN.02
                 ctatgttGATGagcaca 0.994  MEL1.02
```
